# A Heterochromatin Domain Forms Gradually at a New Telomere and Is Dynamic at Stable Telomeres

**DOI:** 10.1128/MCB.00393-17

**Published:** 2018-07-16

**Authors:** Jinyu Wang, Jessica R. Eisenstatt, Julien Audry, Kristen Cornelius, Matthew Shaughnessy, Kathleen L. Berkner, Kurt W. Runge

**Affiliations:** a Department of Genetics and Genome Sciences, Case Western Reserve University, Cleveland, Ohio, USA; b Departments of Immunology and Molecular Genetics, Lerner Research Institute, Cleveland Clinic Foundation, Cleveland, Ohio, USA; c Department of Biochemistry, Case Western Reserve University, Cleveland, Ohio, USA; d Department of Molecular Genetics, The Ohio State University, Columbus, Ohio, USA

**Keywords:** Schizosaccharomyces pombe, chromosome healing, heterochromatin spreading, telomere formation

## Abstract

Heterochromatin domains play important roles in chromosome biology, organismal development, and aging, including centromere function, mammalian female X chromosome inactivation, and senescence-associated heterochromatin foci. In the fission yeast Schizosaccharomyces pombe and metazoans, heterochromatin contains histone H3 that is dimethylated at lysine 9. While factors required for heterochromatin have been identified, the dynamics of heterochromatin formation are poorly understood. Telomeres convert adjacent chromatin into heterochromatin. To form a new heterochromatic region in S. pombe, an inducible DNA double-strand break (DSB) was engineered next to 48 bp of telomere repeats in euchromatin, which caused formation of a new telomere and the establishment and gradual spreading of a new heterochromatin domain. However, spreading was dynamic even after the telomere had reached its stable length, with reporter genes within the heterochromatin domain showing variegated expression. The system also revealed the presence of repeats located near the boundaries of euchromatin and heterochromatin that are oriented to allow the efficient healing of a euchromatic DSB to cap the chromosome end with a new telomere. Telomere formation in S. pombe therefore reveals novel aspects of heterochromatin dynamics and fail-safe mechanisms to repair subtelomeric breaks, with implications for similar processes in metazoan genomes.

## Abstract (precis)

Heterochromatin domains play important roles in chromosome biology, organismal development, and aging, including centromere function, mammalian female X chromosome inactivation, and senescence-associated heterochromatin foci. In the fission yeast Schizosaccharomyces pombe and metazoans, heterochromatin contains histone H3 that is dimethylated at lysine 9.

## INTRODUCTION

A central question in eukaryotic biology is the establishment and maintenance of chromatin domains, i.e., regions of nucleosomal DNA where the histone compositions and spectra of posttranslational modifications are similar. As embryonic cells differentiate, cell-type-specific gene expression is established, in part, by the establishment and maintenance of chromatin domains (e.g., changes in the globin locus in hematopoietic cells and X chromosome inactivation in female mammals [1, 2]). Chromatin domain reorganization also occurs during tumorigenesis as cells transform into rapidly growing cancers (3). Heterochromatin domains, marked in part by nucleosomes with di- and trimethylation of lysine 9 of histone H3 (H3K9me2 or -3), have been intensely studied for their role in chromosome biology. Heterochromatin domains are known for silencing gene expression (4) and can be induced during mammalian cell senescence and aging to form senescence-associated heterochromatin foci containing H3K9me2 (5–7). Heterochromatin also plays an important role at centromeres, the chromosomal structure required for chromosome segregation at mitosis, as centromeric chromatin is flanked by heterochromatin domains that are required for complete function (8–11). While many of the factors required to maintain heterochromatin have been identified, the dynamics of how heterochromatin domains assemble and disassemble are long-standing, major questions that are only now being investigated (12–14).

Telomeres, the physical ends of chromosomes, are a second chromosomal structure bordered by heterochromatin. In yeasts, humans, and many other eukaryotes, telomeres consist of simple DNA repeats bound by specific proteins. These repeats and their associated proteins provide the first discovered essential function of telomeres: “that of sealing the end of the chromosome” (15) and distinguishing it from a double-strand break (DSB) (15, 16). The second essential function is to replace sequences lost due to incomplete replication, which is accomplished by repeat addition via telomerase (17, 18; reviewed in reference 19). Telomeres also alter the adjacent nucleosomal chromatin to silence the expression of nearby genes (20, 21). However, as mutations that eliminate silencing do not cause telomeres to behave as DSBs (20, 22, 23), telomeres perform their essential functions independently of gene silencing. Heterochromatic gene silencing is associated with the presence of H3K9me2 in humans, flies, and the fission yeast S. pombe (24), and S. pombe telomere-associated chromatin has the H3K9me2 modification (22, 23, 25). Thus, S. pombe telomeres provide an ideal model system to study heterochromatin and heterochromatin dynamics.

A major difficulty that impedes the investigation of heterochromatin domain dynamics is the large amount of time between the initiation of domain formation and its analysis. Telomeres have been formed in S. pombe by integrating *in vitro*-constructed telomeric DNA into genomic sites *in vivo*, but 30 population doublings (PDs) or more must pass between the formation of the new telomere and the production of enough cells for chromatin and phenotypic analysis (21). Similar approaches requiring many PDs have followed heterochromatin formation at centromeres and other loci by introducing wild-type genes into mutants defective for heterochromatin assembly (26–29). This approach also converts a mutant cell to a wild-type one, so the levels of cellular chromatin proteins during domain formation are initially different than in wild-type cells. Consequently, additional approaches are needed to study the kinetics of heterochromatin formation and how the H3K9me2 modification spreads from the initiating site into the surrounding chromatin. One hypothesis is that spreading occurs immediately after the initiating site is created and quickly establishes the final heterochromatin domain within one or two generations (as with Sir protein spreading in Saccharomyces cerevisiae [30–32]). Alternatively, the formation of the initiation site, e.g., a functional telomere, may allow spreading over many cell divisions, with the size of the heterochromatin domain gradually increasing over time to form the final state (as suggested for several histone modifications [30] and in S. pombe [14]).

**FIG 1:**
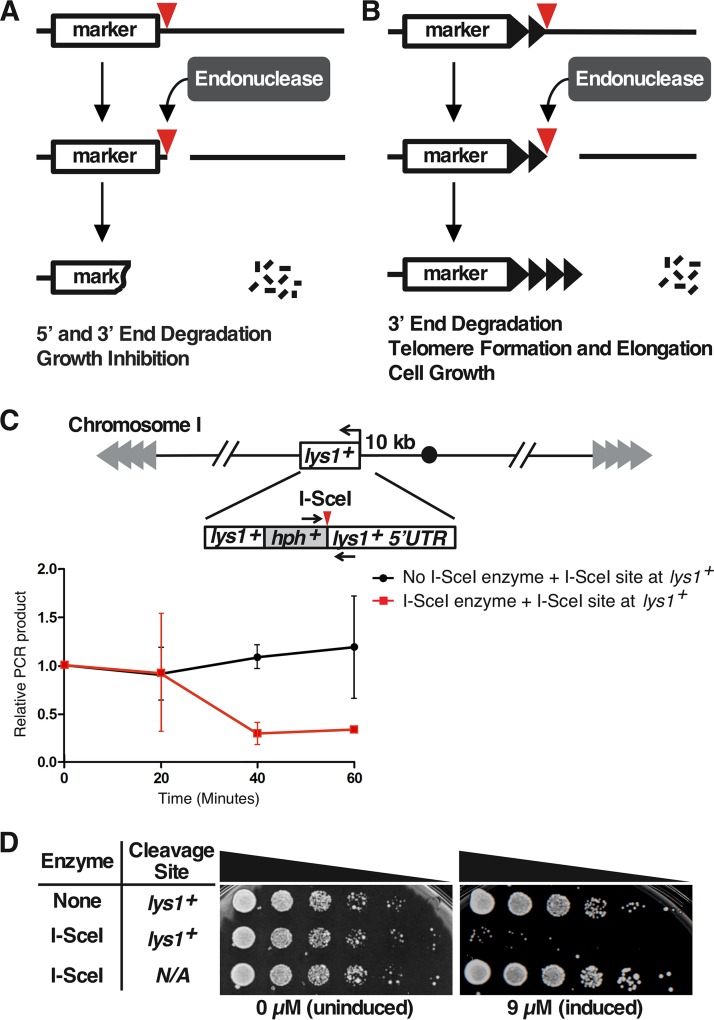
DSB systems and rapid I-SceI DSB formation. (A) Inducible DSB system. A restriction enzyme/endonuclease with no natural sites in the genome is produced in cells from a rapidly inducible promoter. After addition of the inducer and production of the endonuclease, a single site introduced into the genome (red triangle) can be cut to produce a DSB. In the DSB system, the strands both 5′ and 3′ to the endonuclease site are degraded (indicated by short black lines and loss of the marker DNA), and cell growth is inhibited. (B) Inducible telomere formation system. The new DSB exposes telomere repeats (black triangles) to form a new functional telomere that is stable and elongated. If the chromosomal sequences 3′ to the endonuclease site are dispensable, the new functional telomere allows normal cell growth. (C) Rapid induction of an I-SceI-generated DSB. The I-SceI restriction site was inserted into the 5′ UTR of *lys1^+^*, a gene located 10 kb from the centromere of chromosome I. The expression of I-SceI was induced by the addition of ahTET to 9 μM. Cell samples were taken before (0 min) and after ahTET addition at 20-min intervals. Genomic DNA was prepared and assayed for cleavage at the I-SceI site by qPCR using primers across the site (denoted by black arrows) and normalizing to the production of a similarly sized fragment at *his3^+^* (34). The average values from two independent experiments (where each qPCR was performed in triplicate for each experiment), and the standard errors of the mean (SEM) are shown. Note that the assay cannot distinguish between sites that were never cut and those that were cut and then ligated back together with or without mutation of the site. (D) The I-SceI DSB causes growth arrest. Fivefold serial dilutions of cells bearing either the *lys1^+^* allele with or without the I-SceI site or the expression vector with or without the I-SceI gene were spotted onto rich medium with either 0 or 9 μM ahTET. Only cells with both the I-SceI expression vector and the I-SceI site have the capability to produce a DSB, and these cells showed the growth inhibition associated with DSB induction.

An inducible telomere formation system would provide an approach to study the kinetics of heterochromatin formation in wild-type cells. Such systems contain a selectable marker followed by an internal tract of telomere repeats and a unique restriction site or cut site not present elsewhere in the genome. By placing the restriction enzyme or endonuclease gene under the control of a rapidly inducible promoter, one can induce a DSB in a large population of cells to expose the telomere repeats at the new chromosome end (33). In S. cerevisiae and S. pombe, a DSB in the middle of a chromosome normally leads to DNA degradation and growth inhibition (33–35) (Fig. 1A). In contrast, a telomere formation system in S. cerevisiae and mammalian cells has shown that a DSB that exposes telomere repeats is immediately converted into a short, functional telomere that is not degraded (33, 36–38) (Fig. 1B). The S. cerevisiae telomere formation system has yielded important insights into the roles of telomerase, telomere binding proteins, DNA polymerases, and DNA damage proteins in telomere elongation (reviewed in reference 39). However, S. cerevisiae lacks the H3K9me2 modification system, so its use in modeling the kinetics of heterochromatin spreading that occurs in metazoans is limited.

S. pombe is a useful model for studying the H3K9me2 heterochromatin system (40), but a telomere formation system was previously not feasible owing to the lack of a method to rapidly induce a DSB. Two different rapidly inducible systems have recently been established by Watson et al. using the HO endonuclease (41) and by ourselves using I-PpoI endonuclease (34). Unfortunately, neither system was well suited to inducing telomere formation. The HO system uses an *urg1^+^* promoter that is induced by the addition of uracil, which interferes with the use of the *ura4^+^* selectable marker. Expression of the *ura4^+^* gene can be selected for or against, which allows the facile monitoring of expression by cell growth, and has been a mainstay of gene silencing studies (21, 42, 43). Our I-PpoI system avoids this *urg1^+^* limitation by using an anhydrotetracycline (ahTET)-inducible promoter, but I-PpoI cuts in the rDNA of almost all eukaryotes, so strains bearing mutated rDNA repeats must be used. We therefore designed a new method to rapidly induce a single DSB in the S. pombe genome and used it to create a telomere formation system. Telomere formation was induced in a population of cells to follow heterochromatin formation in real time. While a functional telomere that was distinct from an unstable DSB formed immediately, the H3K9me2 modification spread slowly from the new telomere over several generations. Surprisingly, chromatin immunoprecipitation (ChIP) and gene expression reporters revealed that the extent of spreading varied with growth conditions and over time even when the lengths of the telomere repeat tracts were constant. Thus, the established heterochromatin domain was surprisingly dynamic. We also discovered that a subtelomeric DSB in the euchromatin that lacks telomere repeats was rapidly healed with high efficiency, in contrast to breaks in the middle of the chromosome. Therefore, the structure of the S. pombe genome contains an unanticipated fail-safe mechanism to rescue telomere loss. These results in S. pombe suggest similar novel processes may also occur at metazoan telomeres and heterochromatin domains.

## RESULTS

### The S. pombe telomere formation system.

**FIG 2:**
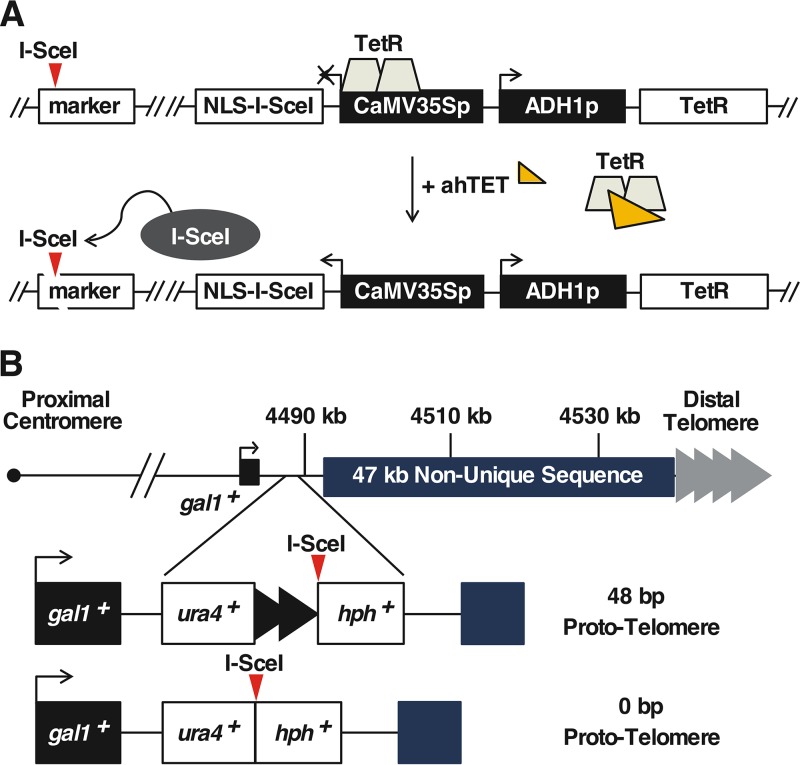
I-SceI telomere formation system. (A) The I-SceI endonuclease was expressed from the TetR-controlled CaMV35S promoter in a cassette that also expresses TetR. The addition of ahTET induces I-SceI expression, which then cuts at sites introduced into the genome (red triangles). (B) The 48-bp prototelomere contains the *ura4^+^* gene followed by 48 bp of telomere repeats (black triangles) and the hygromycin resistance marker (*hph^+^*), while the 0-bp prototelomere control lacks the telomere repeats. Both cassettes were inserted into the unique DNA 3′ of the *gal1^+^* gene. The S. pombe endogenous telomere repeat tracts are indicated by gray triangles.

We first developed an inducible DSB system in S. pombe using the I-SceI homing endonuclease. While I-SceI has no endogenous sites in the S. pombe genome (44) and has the advantages described above, it has the disadvantage of inefficient and slow cutting (45, 46). We therefore designed an I-SceI gene with preferred S. pombe codons (47) and two nuclear localization signals (NLS) at the N terminus to enhance expression and genomic DNA cleavage. This I-SceI variant was expressed from a TetR-repressed promoter, which allows expression of the desired gene after addition of ahTET (Fig. 2A). Cutting efficiency was tested in a strain with the I-SceI site at a marker gene near the centromere of chromosome I, *lys1^+^* (Fig. 1C). Most I-SceI sites were cut within 40 min of induction of I-SceI expression (Fig. 1C). When plated on inducing medium, the strain expressing I-SceI and containing a site at *lys1^+^* showed a severe growth defect (Fig. 1D), as has been seen with other strains that continuously induce a DSB (34, 48–50).

Two “prototelomere” cassettes were created that contained either 48 bp or 0 bp of S. pombe telomere repeat sequence, an I-SceI site, and two flanking selectable markers (Fig. 2B). Cleavage at the I-SceI site should expose the telomere repeats and cause loss of the distal marker and chromosome end. Consequently, to yield viable cells for analysis, the lost region had to be dispensable and the 48-bp telomere repeats must form a new functional telomere. We therefore chose a site in the 2-kb region 3′ to the *gal1^+^* gene on the right end of chromosome II (IIR), because this region is unique in the genome and borders a 47-kb subtelomere-containing sequence that is repeated at both ends of chromosomes I and II (44, 51, 52). Cells that have lost most of these subtelomeric sequences are viable (52). In addition, *gal1^+^* and each gene in the 86-kb region 5′ to *gal1^+^* are not required for growth. Therefore, induction of I-SceI cleavage in the 2-kb region 3′ to *gal1^+^* could cause loss of dispensable chromosomal sequences and allow the formation of a large heterochromatic domain near the prototelomere and still produce viable cells.

### A functional short telomere forms after I-SceI cleavage.

**FIG 3:**
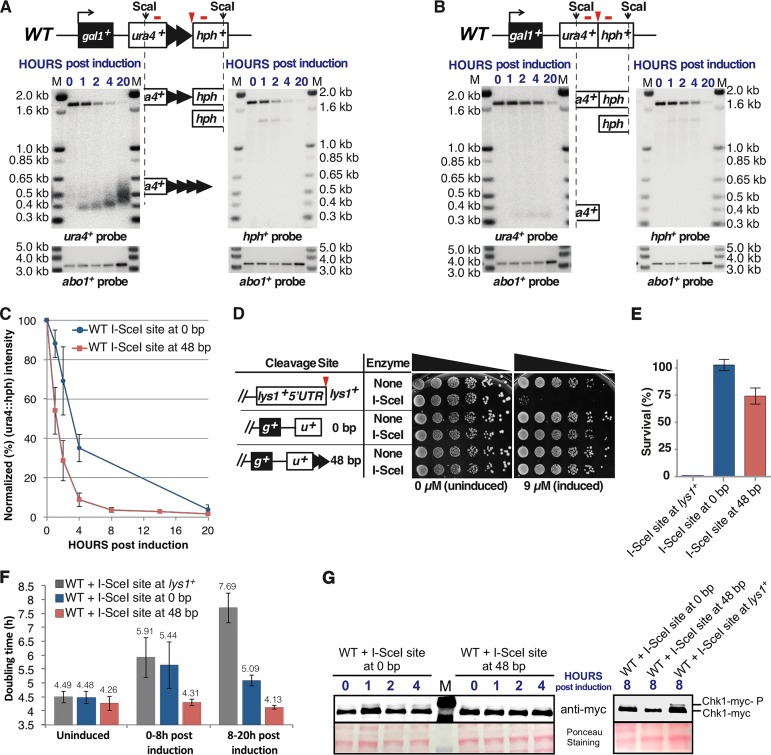
I-SceI cleavage converts the 48-bp prototelomere to a telomere. (A) Exponentially growing cells bearing the 48-bp prototelomere and the I-SceI expression cassette were treated with ahTET (9 μM final concentration), and aliquots were taken either prior to induction (0 h) or after induction (1 to 20 h). *WT* signifies the wild-type genomic background for the telomere formation strains (e.g., YJRE210 [Table 1]). Genomic DNA was digested with ScaI and analyzed by Southern analysis using probes for *ura4^+^* or *hph^+^* (denoted by red bars above each locus). The I-SceI site is marked by a red triangle. The prototelomere fragment is rapidly converted to the smaller *ura4^+^* and *hph^+^* fragments. The I-SceI-cleaved ScaI-*ura4^+^* and *hph^+^*-ScaI bands are indicated by partial ideograms of the original diagram of the prototelomere. The numbers in blue above the blot represent the hours postinduction. As a control for loading, the blots were rehybridized with an *abo1^+^* probe, shown below the Southern blot. Molecular size standards are shown (lanes M). (B) Cells bearing the 0-bp prototelomere cassette were treated and analyzed as for panel A. (C) Normalized intensities of the 1.8-kb uncut *ura4*^+^::*hph^+^* band from cells bearing the I-SceI expression cassette and the 48-bp prototelomere or the 0-bp prototelomere. Normalization was the average of the Typhoon Imager signal for either *ura4^+^* or *hph^+^* divided by the *abo1^+^* signal for the respective lane. The error bars show SEM from triplicate assays. (D) Serial 5-fold dilutions of cells bearing an I-SceI site at *lys1^+^* and the 48- and 0-bp prototelomere cassettes were spotted onto minimal medium that lacked or had ahTET (9 μM). The diagrams show one side of each cleavage site (*g^+^*, *gal1^+^*; *u^+^*, *ura4^+^*). (E) Quantitation of survival of the strains shown in panel D after induction of I-SceI. Survival of both the 0-bp and 48-bp prototelomere strains was significantly different than that of the strain bearing the I-SceI site at *lys1^+^* (*P* < 0.01; *t* test). The 0-bp and 48-bp strains were not significantly different (*P* = 0.09; *t* test). The error bars show SEM from duplicate assays. (F) Doubling times of 3 independently induced cultures obtained by determining cell concentration using a hemocytometer. More than 1,000 cells were counted for each genetic construction (the I-SceI expression cassette with an I-SceI site at *lys1^+^* or at the 0-bp or 48-bp prototelomere). The doubling times prior to induction (uninduced), 0 to 8 h postinduction, and 8 to 20 h postinduction are shown above the bars. The error bars show standard deviations from 3 independent assays. (G) Western analysis of Chk1-myc in cells bearing the I-SceI expression cassette and the I-SceI site at *lys1^+^* or the 48-bp and 0-bp prototelomere cassettes. Cell samples were taken prior to induction (0 h) or after the addition of ahTET to 9 μM (1 to 8 h). Whole-cell extracts were then analyzed by Western blotting with an antibody directed against the myc epitope tag at the C terminus of Chk1. The 0.1% Ponceau S (5% acetic acid) staining of the membrane shows the relative amounts of protein loaded.

**FIG 4:**
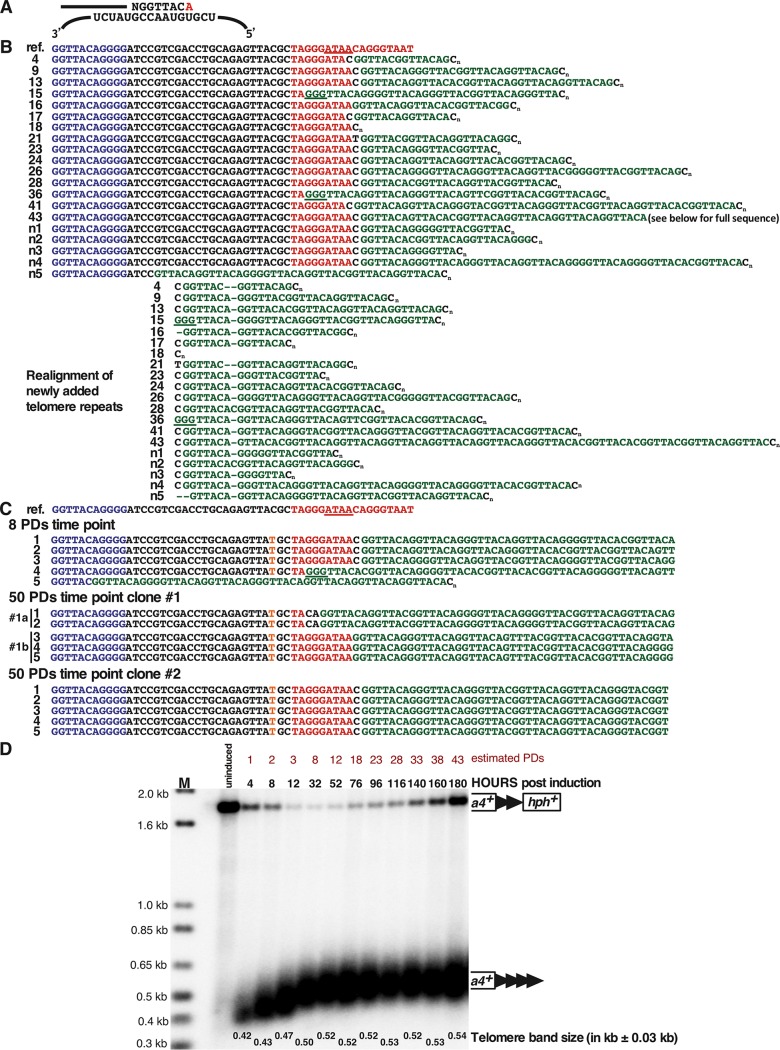
Analysis of the newly added telomere repeats at the 48-bp prototelomere. (A) Sequences added by S. pombe telomerase in the first round of synthesis. The top line represents a DNA strand further elongated by telomerase, where the added GGTTACA sequence hybridizes to the telomerase RNA template (bottom strand) (53). The red A at the 3′ end may or may not be added. (B) Sequences of newly added telomere repeats from cells at the ∼1-PD time point (4.2 h postinduction). The top row (ref.) shows the reference sequence of the 48-bp prototelomere. The sequences in blue represent part of the 48 bp of telomere repeats, black is the polylinker sequence, red is the I-SceI site, with the underlined red bases representing the overhang after I-SceI cleavage, and green is the newly added telomere repeats. C_n_ indicates the oligo(dC) added during the telomere PCR amplification (87). The 20 rows below are sequences from 20 individual clones collected at the ∼1-PD time point. The realigned sequences separate the first newly added telomere repeat, NGGTTAC(A), of all the telomeres from the 3′ sequences. The underlined GGG sequences may have been part of the original I-SceI site or added by telomerase. Interestingly, all telomere repeat addition was to the I-SceI site or polylinker sequences, similar to telomerase-mediated repeat addition in S. cerevisiae (54, 55, 94) and mammalian cells (38, 56). (C) Telomere sequences cloned from the ∼8-PD time point or different clones from the ∼50-PD time point. These fully elongated telomeres still retain the polylinker and I-SceI site in all but one case, indicating that this conformation forms a stable telomere. A C-to-T point mutation in the polylinker sequence in the clones is highlighted in orange. The ∼50-PD clone 1 appears to be a mixture of two clones, as two sequences (1a and 1b) were rescued from the culture. Only the telomere repeat sequences closest to the addition site are shown. (D) Telomere repeat tracts are fully elongated by ∼8 PDs after prototelomere cleavage. After induction of I-SceI, cells were grown for multiple PDs in liquid culture with 9 μM ahTET by serial dilution, and samples from different time points were processed for Southern blotting using *ura4^+^* as a probe, as for Fig. 3A. The modal terminal restriction fragment (TRF) sizes of the newly formed telomere (*ura4^+^* telomere repeat band) after induction was measured at the most intense hybridizing point in the band. Band sizes on these blots vary by approximately ±0.03 kb. Molecular size standards are shown (lane M). The data reveal that cells with an uncleaved prototelomere had a growth advantage over cells with the new telomere, so that the cells with the uncleaved prototelomere increased in proportion during continuous growth. The uncleaved prototelomeres most likely resulted from cassettes that were cut and healed by a DNA repair event that eliminated the I-SceI site.

**FIG 5:**
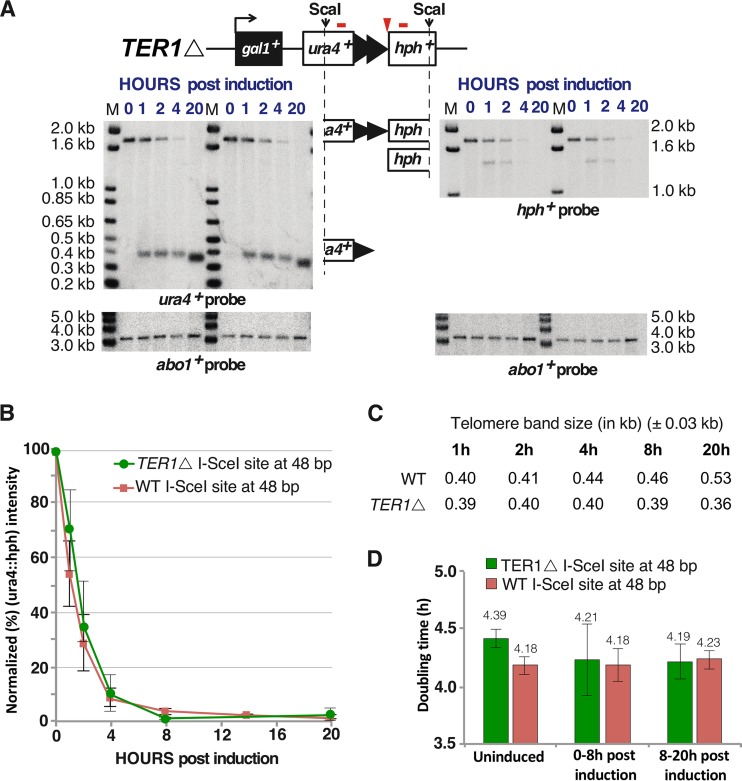
Telomerase-null cells form stable telomeres without elongation after induction of chromosome breakage. (A) Two independent telomerase-null (*TER1*Δ) mutants were separately induced with ahTET and analyzed as for Fig. 3A. The *ura4^+^* and *hph^+^* ScaI bands are indicated by partial ideograms of the uncut prototelomere diagram. (B) Efficiency of prototelomere cleavage, determined as for Fig. 3C. Results from *TER1*Δ and wild-type (WT) strains are shown. The error bars show SEM from duplicate assays for the *TER1*Δ strain and triplicate assays for the WT strain. (C) The modal TRF sizes of the newly formed telomere after induction were measured as for Fig. 4C. Band sizes on these blots vary by approximately ±0.03 kb. (D) Doubling times of wild-type or *TER1*Δ cells bearing the 48-bp prototelomere and I-SceI expression cassette prior to ahTET induction (uninduced), 0 to 8 h postinduction, and 8 to 20 h postinduction (as determined by the OD_600_).

Telomere formation was induced in S. pombe cells containing the 48-bp prototelomere by expressing I-SceI and monitoring the fate of the *ura4^+^* and *hph^+^* prototelomere fragments by Southern analysis (Fig. 3A). The uncut *ura4^+^*-telomere repeats-*hph^+^* band was visible prior to induction of I-SceI and was replaced by the I-SceI-cleaved *ura4^+^* and *hph^+^* bands over time. Cleavage was efficient, as over 90% of the *ura4^+^ hph^+^* fragments were cleaved within 4 h (Fig. 3C), which was less than 1 PD under these conditions. The *ura4^+^* fragment was stable and increased in size and heterogeneity during the experiment, as expected for the elongation of the exposed 48-bp telomere repeats (Fig. 3A). Elongation was almost certainly by telomerase, as sequencing of the telomere fragments revealed that, in all but one case, addition of new telomeric repeats to the I-SceI-cleaved prototelomere occurred (Fig. 4B), consistent with telomerase-mediated addition events in S. cerevisiae and mammalian cells (38, 53–56). When telomere formation was performed in cells lacking telomerase RNA, the newly formed telomere was stable but not elongated (Fig. 5). In contrast, the *hph^+^* fragment was rapidly degraded (Fig. 3A). Thus, formation of a telomere, the stable structure that “seals” the end of the chromosome (15), occurs at the earliest time point tested and is independent of telomerase activity. Following elongation of the telomere repeats for over 50 PDs revealed that the telomere repeat tracts were stably maintained (Fig. 4C) and reached their equilibrium final lengths by ∼8 PDs (Fig. 4D). We noted that some cells with uncut telomeres regrew during this serial dilution experiment (Fig. 4D), and a method to eliminate cells with uncut prototelomeres after a few PDs was instituted for the ChIP experiments discussed below. The initial stability and subsequent elongation of the *ura4*^+^ 48-bp telomere band, therefore, show that this fragment rapidly acquired the essential telomeric functions of end capping and end replication after I-SceI cleavage and behaved the same as short functional telomeres at chromosome ends (e.g., newly formed S. cerevisiae telomeres and telomeres of cells lacking Tel1, MRX, or Ku [20, 57]).

The I-SceI-induced DSB at the 0-bp prototelomere had a fate notably different from that of the 48-bp prototelomere. The 0-bp prototelomere strain displayed rapid cutting, as demonstrated by the disappearance of the *ura4^+^ hph^+^* fragment and degradation of both I-SceI-generated terminal fragments by 20 h postinduction (Fig. 3B and C). Thus, I-SceI cutting at this locus was efficient, and both sides of the DSB at the 0-bp prototelomere were unstable.

### The genomic organization of S. pombe allows the efficient healing of subtelomeric DSBs.

Double-strand breaks cause growth arrest in S. pombe, S. cerevisiae, human cells, and other model systems, while telomeres do not (34, 50; reviewed in reference 39). We therefore tested the effect of I-SceI cleavage at centromeric *lys1^+^* and the subtelomeric 48-bp and 0-bp prototelomeres. As expected, cleavage at *lys1^+^* greatly impaired growth (Fig. 3D and E). In contrast, cleavage at the 48-bp prototelomere, which formed a telomere and lost subtelomeric repeated sequences, showed no detectable growth inhibition. Surprisingly, cells containing the 0-bp prototelomere cassette also showed very little growth inhibition, with ∼100% of the cells surviving (Fig. 3D and E), even though the *ura4^+^* fragment had been degraded in these cells (Fig. 3B). The mechanism allowing this survival was unclear, because the DSB occurred in unique sequence, not in the sequences repeated in four telomeres.

**FIG 6:**
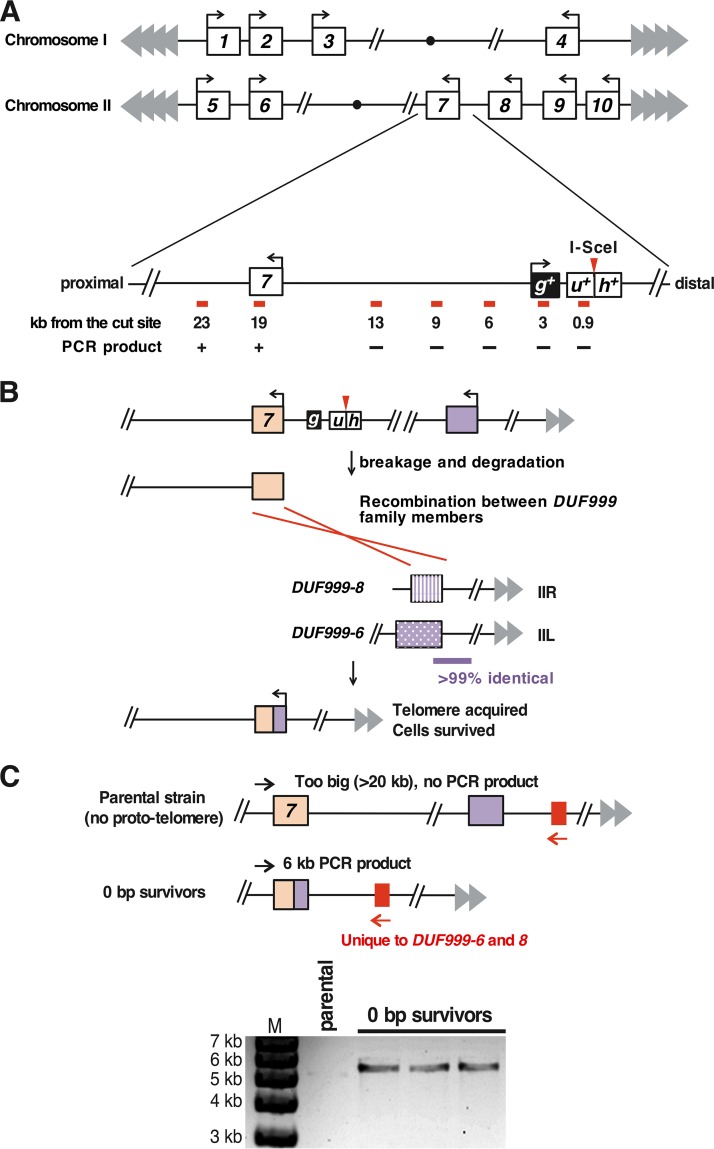
The genome organization of S. pombe allows efficient healing of the 0-bp prototelomere. (A) Map of the *DUF999* gene family on chromosomes I and II. All 10 genes from the family are in the same orientation, with transcription toward the centromere (circles). The chromosome III subtelomeres consist of scores of rRNA gene repeats (44) and are not shown. The region near the *DUF999-7* gene (boxed 7) that is just internal to the 0-bp prototelomere insertion site is expanded, showing the relative position of the prototelomere and the distances of different primer pairs (shown as red bars) from the I-SceI site. A plus indicates that a PCR product was obtained from each of the three surviving cleaved 0-bp prototelomere strains tested, and a minus indicates that a product was not obtained. The *DUF999-7* gene was the gene closest to the degradation endpoint. (B) Hypothesis to explain how the *DUF999* gene family can provide a backup mechanism to rescue a DSB near the subtelomere. After induction of a DSB at the 0-bp prototelomere, DNA is degraded at both ends (Fig. 3B). The generation of degraded DNA in the *DUF999-7* gene can produce a DSB that can undergo recombination with other *DUF999* genes (purple boxes) to acquire a new telomere. To test this hypothesis, we performed inverse-circle PCR (see Materials and Methods) and determined the sequences that had been fused to the *DUF999-7* gene. We found a recombination donor that could be from *DUF999-3*, -*6*, or -*8*. (C) PCR to confirm the recombination event. *DUF999-6* and -*8* have a unique region (red box) that is absent from the *DUF999-3* gene region. PCR using a primer specific to this region (red arrows) and a unique primer at *DUF999*-7 (black arrows) revealed that the three strains that survived the induction of the DSB (the 0-bp survivors) were generated from the recombination between the *DUF999-7* gene and *DUF999-6* or -*8* gene. The sequence between *DUF999-6* and its telomere is nearly identical to the sequence between *DUF999-8* and its telomere, and thus, the recombination event that rescued the DSB was not pursued further.

To determine what process allowed the efficient growth of cells bearing the DSB formed at the 0-bp prototelomere, we determined the chromosomal structures of three independent survivors. Phenotypic and genomic characterization revealed that the survivors had lost the *hph^+^* gene and almost 19 kb of DNA internal to the I-SceI cleavage site. The degradation endpoint retained the *DUF999* protein family 7 gene (*DUF999-7*), a member of a gene family near the telomeres of chromosomes I and II in which all the genes are transcribed toward the centromere (Fig. 6A). We hypothesized that nucleolytic degradation from the I-SceI site to the *DUF999-7* gene would allow recombination between gene family members to add a functional chromosomal end to IIR (Fig. 6B), as recombination between repeats is known to be efficient enough to account for this high level of survival (58). To test this hypothesis, we determined the sequences adjacent to *DUF999-7* in the survivor strains and found sequences indicating recombination with *DUF999-8* on IIR or *DUF999-6* on the left end of chromosome II (IIL) (Fig. 6B and C). As the sequences from the *DUF999-8* and *DUF999-6* genes to their respective telomeres were almost identical (51, 52), the specific telomere captured by the DSB was not determined. Therefore, the S. pombe genome is structured to rapidly and efficiently heal DSBs near subtelomeres and to maintain cell viability.

### Cleavage of the 48-bp prototelomere does not impair cell growth or cause Chk1 phosphorylation.

**FIG 7:**
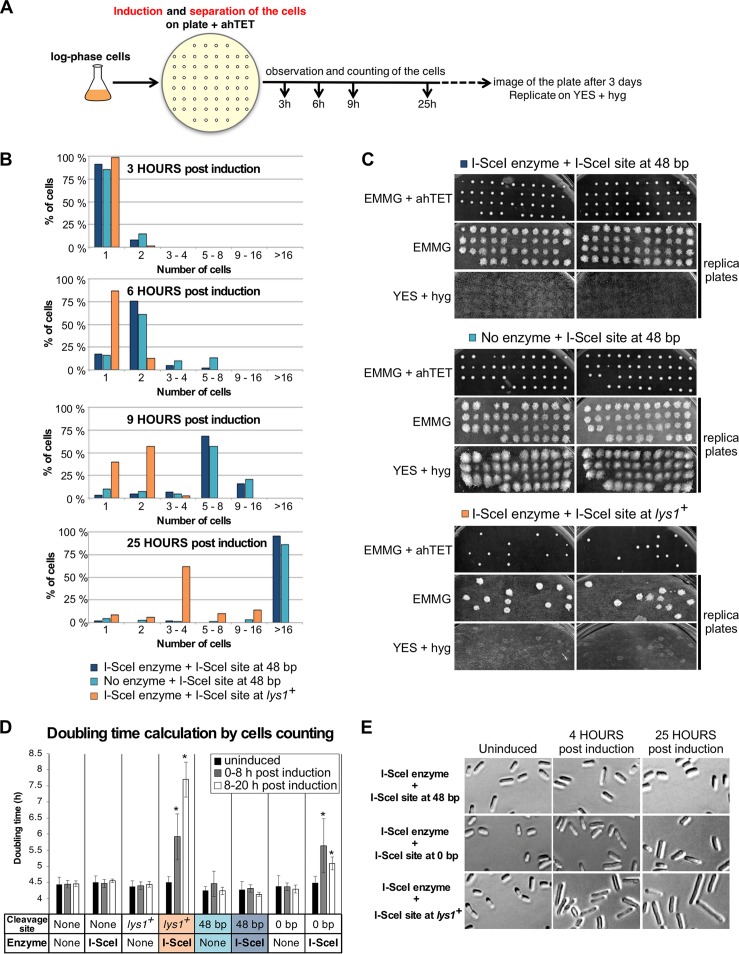
Cleaving the 48-bp prototelomere does not induce a cell cycle pause. (A) Experimental design. Telomere formation was induced on synthetic nonselective plates supplemented with 9 μM ahTET. Single cells (40 to 44) were micromanipulated onto a grid, and cell numbers were observed after incubation for 3, 6, 9, and 25 h at 32°C. The plates were incubated for an additional 3 days, scanned, and then replicated on nonselective plates and on YES-plus-hygromycin plates, which were then grown for 3 days at 32°C and scanned. (B) Numbers of cells of different strains at 3 to 25 h postinduction. Cells bearing the I-SceI site at 48-bp prototelomeres with or without the I-SceI expression cassette or cells with an I-SceI site at *lys1^+^* and the I-SceI expression cassette were assayed. (C) Cells from panel B were grown for 3 days at 32°C, replica plated to the indicated media, and grown for 3 more days at 32°C. Images of the original plate (EMMG plus ahTET) and the two replica plates are shown for each strain. (D) Doubling times of the strains with the 48-bp or 0-bp prototelomere or the *lys1^+^* I-SceI site with or without the I-SceI expression cassette. Doubling times from three independently induced cultures were calculated by counting cells using a hemocytometer at the indicated time points. More than 1,000 cells were counted for each genetic construction. The doubling times of different strains were compared by *t* test to that of the wild-type strain lacking an I-SceI site and the I-SceI expression cassette. Significant differences (*P* < 0.05) are marked by asterisks. (E) Representative images of cells bearing the I-SceI site at the 48-bp or 0-bp prototelomere or at *lys1^+^*. Growing cells prior to induction (uninduced) or 4 or 25 h postinduction were photographed at the same magnification to compare cell sizes after I-SceI cuts at the different loci. The strain with a *lys1^+^* I-SceI site shows the elongation phenotype characteristic of G_2_/M arrest due to DNA damage.

The cellular responses to I-SceI cleavage at *lys1*^+^ versus the 48- and 0-bp prototelomeres suggested different levels of checkpoint activation in response to the induced DSB. Previous work in S. cerevisiae using a construct similar to the 48-bp prototelomere showed that the induced DSB caused a short growth delay that was distinguishable from the growth with and without a chromosomal DSB, a phenomenon called the “telomere anticheckpoint” (36, 37, 59). To determine if the S. pombe system showed telomere anticheckpoint activity, the growth rates of single cells bearing the 48-bp prototelomere, with or without the I-SceI expression cassette, and cells bearing the I-SceI expression cassette and the *lys1*^+^ I-SceI site were determined after induction. Exponential-phase cells were plated on nonselective medium with anhydrotetracycline (ahTET), and single cells were micromanipulated onto a grid for monitoring of cell growth by microscopy (Fig. 7A). In two separate experiments, cells bearing the 48-bp prototelomere grew at the same rate whether or not the I-SceI expression cassette was present (Fig. 7B). After these cells grew into colonies, they were assayed by replica plating for cleavage at the I-SceI site and loss of the distal hygromycin resistance gene. All of the colonies with the I-SceI expression cassette were hygromycin sensitive, indicating that I-SceI cleavage and telomere formation occurred as in the liquid cultures (Fig. 3A). Cells lacking the I-SceI expression cassette were hygromycin resistant, indicating lack of cleavage. In marked contrast, cells bearing the *lys1*^+^ I-SceI site and I-SceI cassette showed a growth delay, with the majority having completed only 2 or 3 cell divisions when the cells with the 48-bp prototelomere had completed more than 16 divisions (Fig. 7B, 25 h). A fraction of the *lys1*^+^ I-SceI cells did form hygromycin-sensitive colonies (∼25%) (Fig. 7C), suggesting that some portion of the *hph^+^* gene and the adjacent I-SceI site (Fig. 1C) had been deleted. These results indicate that the I-SceI cassette was quickly expressed upon plating on the ahTET plates and that I-SceI cleavage at the 48-bp prototelomere did not induce a detectable growth delay.

The single-cell growth phenotypes suggested that the cleaved 48-bp prototelomere did not activate the DNA damage cell cycle checkpoints while the DSB at *lys1*^+^ did. Both cleavages were associated with DNA degradation (Fig. 3A), suggesting that the cut prototelomere had anticheckpoint activity. Activation of the DNA damage checkpoint in S. pombe causes cells to arrest at G_2_/M, grow into elongated cells, and phosphorylate the kinase Chk1 (60, 61). Therefore, cells bearing the 48-bp prototelomere and *lys1*^+^ I-SceI site with and without the I-SceI expression cassette were induced in liquid culture to determine the doubling time, the cell morphology, and the status of Chk1 phosphorylation. Cleavage at the 0-bp prototelomere also results in DNA degradation (Fig. 6) with no loss of viability (Fig. 3D and E), and these cells were also analyzed.

Both strains that have and strains that lack the I-SceI expression were assayed for growth. All the cells lacking I-SceI expression had similar doubling times in uninduced cultures, during the first 8 h postinduction and the last 8 to 20 h postinduction (Fig. 7D). Cells bearing the 48-bp prototelomere and the I-SceI cassette had the same doubling time after I-SceI induction. Inducing I-SceI expression in cells bearing the *lys1* I-SceI site caused cell doubling times to increase over the first 8 h postinduction and to increase further in the subsequent 8 to 20 h, suggesting checkpoint activation (Fig. 3F). The 0-bp prototelomere showed an intermediate phenotype, with an increase in doubling time in the first 8-h period that decreased in the next 8 to 20 h. Cell morphologies mirrored these growth rate phenotypes in that the cells with the 48-bp prototelomere had the same cell size in uninduced and induced cultures, cells with the 0-bp prototelomere had slightly longer cells after induction, and cells with the *lys1^+^* I-SceI cassette formed elongated cells characteristic of a G_2_/M cell cycle delay (Fig. 7E). The level of checkpoint activation can also be monitored by the level of phosphorylated Chk1, which migrates as a slower band than Chk1 on SDS-PAGE gels (60). No increased Chk1 phosphorylation was detectable in cells with the 48-bp prototelomere during the first 8 h after induction (Fig. 3G). In cells with the 0-bp telomere, a faint band of phosphorylated Chk1 was detectable during the first 4 h postinduction. Finally, the induced DSB at *lys1*^+^, which caused a strong growth delay in the single-cell analysis (Fig. 7B), showed the largest amount of Chk1 phosphorylation at 8 h (Fig. 3G). The sum of these data demonstrates that the DSB at *lys1*^+^ causes strong checkpoint activation; the 0-bp prototelomere induces weak, transient checkpoint activation; and the cleaved 48-bp prototelomere has telomere anticheckpoint activity.

### Telomere formation initiates silencing of *ura4^+^*.

**FIG 8:**
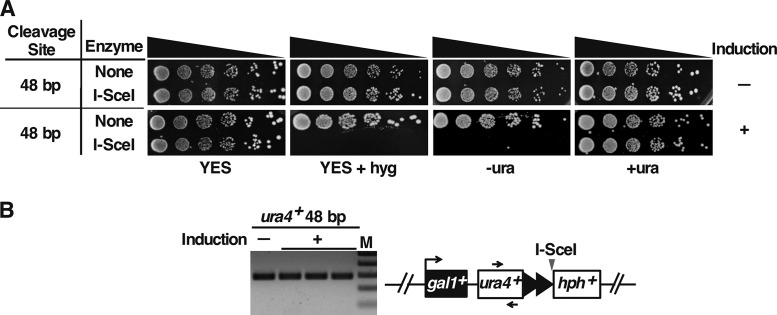
Telomeric *ura4*^+^ is silenced. (A) Growth of cells containing the 48-bp prototelomere before and after 20 h of I-SceI induction was assessed by spotting 5-fold serial dilutions of cells onto rich-medium plates (YES [81]) with and without hygromycin (hyg), or synthetic EMMG medium with (+ura) or without (−ura) uracil. (B) The presence of the *ura4^+^* gene in untreated 48-bp prototelomere cells prior to induction (−) or in three independent Ura^–^ hygromycin-sensitive colonies derived from ahTET-treated cells (+) was tested by PCR. Primers are indicated by arrows.

To test silencing at a newly formed telomere, telomere formation was induced and the cells were subsequently assessed for expression of the *ura4^+^* marker. Cells in which transcription of *ura4^+^* is silenced, such as by placing *ura4^+^* near a newly formed telomere, are unable to grow on media lacking uracil (21, 23, 62). Cells were therefore induced for I-SceI expression overnight prior to plating on rich and selective media (Fig. 8A). After induction, strains with the 48-bp prototelomere grew poorly on medium containing hygromycin, indicating loss of the 3′ *hph^+^* fragment (Fig. 3A), or on medium lacking uracil. However, the *ura4^+^* gene could be amplified by PCR from these Ura^−^ colonies (Fig. 8B), and a PCR product of the entire *ura4^+^* gene had the wild-type sequence (not shown). Therefore, establishing a new telomere silenced expression of the adjacent *ura4^+^* gene.

**FIG 9:**
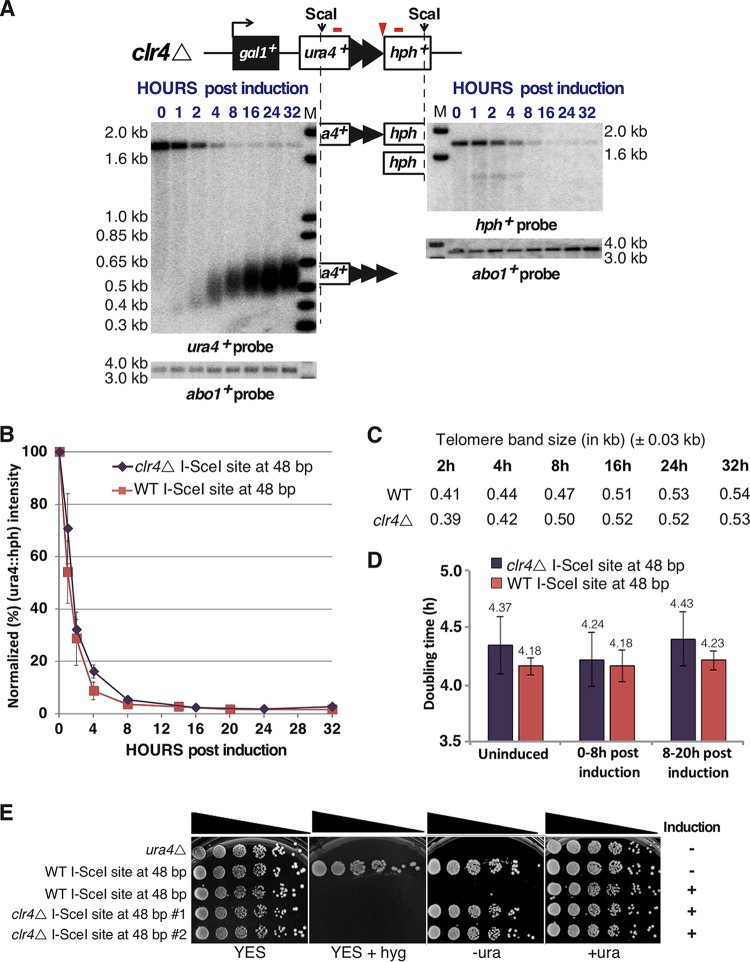
Heterochromatin establishment in a newly formed telomere is independent of its elongation. Clr4 is the only histone H3 lysine 9 methyltransferase and is required for heterochromatin. (A) Exponentially growing wild-type and *clr4*Δ cells bearing the 48-bp prototelomere and the I-SceI expression cassette were treated with ahTET (9 μM final concentration), and aliquots were taken either prior to treatment (0 h) or after treatment (1 to 32 h), following the approach used for Fig. 3A. Blots from the *clr4*Δ cells are shown. The wild-type cells gave results nearly identical to those in Fig. 3A. (B) Efficiency of *ura4^+^*::*hph^+^* cleavage, as shown in Fig. 3C. The error bars show SEM from duplicate assays for *clr4*Δ and triplicate assays for the wild type (WT). (C) Telomere elongation is nearly identical in wild-type and *clr4*Δ backgrounds. The modal TRF sizes of the newly formed telomere after induction were measured as for Fig. 4C. Band sizes on these blots vary by approximately ±0.03 kb. WT sizes are from the formation experiment shown in Fig. 3A. *clr4*Δ sizes are from the formation experiment shown in panel A. (D) Doubling times of wild-type or *clr4*Δ cells bearing the 48-bp prototelomere and the I-SceI expression cassette prior to ahTET induction (uninduced), 0 to 8 h post induction, and 8 to 20 h postinduction (as determined by the OD_600_). (E) The *clr4*Δ mutation eliminated telomeric silencing. Growth of wild-type cells (*ura4^−^*) or wild-type or *clr4*Δ cells bearing a 48-bp prototelomere before (−) and after (+) 20 h of induction was assessed by spotting 5-fold serial dilutions of cells onto rich-medium plates (YES) without and with hygromycin (hyg) or onto synthetic medium without (−ura) or with (+ura) uracil.

To determine if heterochromatin impacted telomere formation, the *clr4^+^* gene encoding the H3 lysine 9 methyltransferase was deleted from the strain containing the 48-bp prototelomere and I-SceI expression cassette. The *clr4*Δ mutation resulted in expression of *ura4^+^* at a fully formed telomere in two independent isolates (Fig. 9E), which were examined for telomere formation. Upon I-SceI induction, cleavage of the 48-bp prototelomere, elongation of the telomere repeats, and the final size of the telomere band were nearly identical in the wild-type and *clr4*Δ strains (Fig. 9A to C). The *clr4*Δ strain had a doubling time slightly longer than that of wild-type cells, and telomere formation did not alter this differential (Fig. 9D). Thus, the absence of Clr4 had no detectable effect on telomere formation and elongation kinetics, indicating that the two processes are independent.

### The H3K9me2 heterochromatin mark spreads gradually after telomere formation and is highly variable at full-length telomeres.

**FIG 10:**
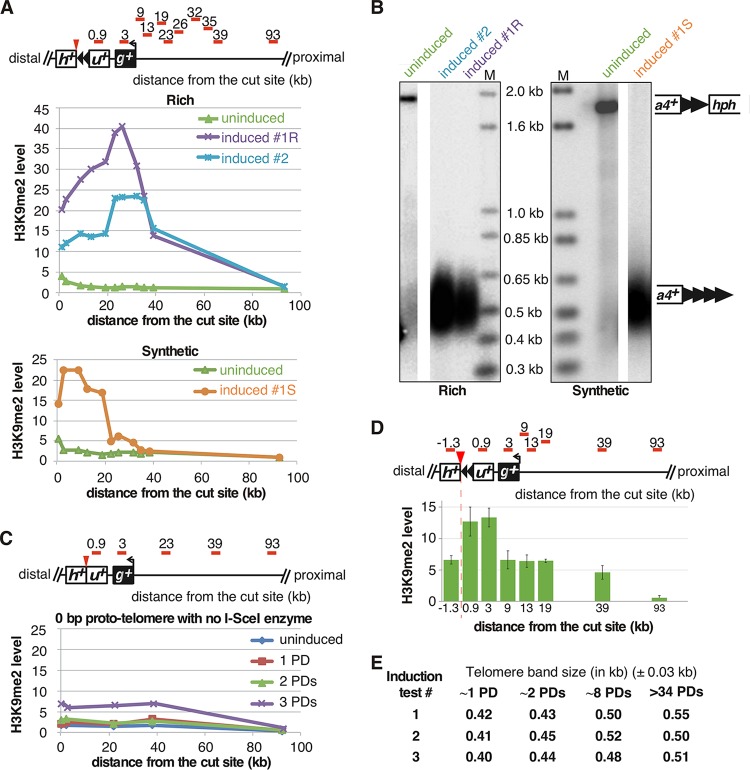
Different telomeric H3K9me2 domains form in cells with telomeres of similar sizes. (A) Level of histone H3K9me2 enrichment at each locus (relative primer locations are shown as red bars in the diagram at the top). The purple and cyan lines (top graph) represent two individual clones of 48-bp prototelomere grown in rich medium (YES plus 3% glucose) for 50 PDs after induction, which both peak between 19 and 39 kb from the cut site. In contrast, cells grown in synthetic medium (EMMG with uracil plus 2% glucose) (bottom graph) have the highest H3K9me2 level at a locus much closer to the newly formed telomere. The green lines (both graphs) represent the uninduced 48-bp prototelomere, which shows a small increase next to the telomere repeat tracts in the prototelomere. (B) The established telomeres have similar lengths in cells from rich (left) or synthetic (right) medium. The lanes with DNA from cells in panel A are labeled at the top. Southern analysis used the *ura4^+^* probe, as for Fig. 3A. Molecular size standards are shown (lanes M). (C) 0-bp control cells do not have increased H3K9me2 levels after induction. The levels of histone H3K9me2 enrichment at each locus are shown as in panel A. A schematic of the 48-bp prototelomere shows the locations of the 0.9- and 3-kb probes in *ura4^+^* and *gal1^+^*, respectively. (D) The H3K9me2 modification extends into the *hph^+^* gene in the uncut telomere. (Top) Relative positions of qPCR primers (red bars) for the assays. (Bottom) H3K9me2 levels by qPCR. The error bars are SEM from triplicate assays. (E) Telomere elongation is nearly identical in independent telomere formation experiments. The modal TRF sizes of the newly formed telomere after induction were measured as for Fig. 4C. Band sizes on these blots vary by approximately ±0.03 kb. The different inductions are, respectively, from the experiments shown in Fig. 11C and 12C, 13 and 11D, and 12D.

To further characterize *ura4^+^* silencing, the levels of a heterochromatin-specific histone modification, H3K9me2, were monitored near the established telomere by ChIP-quantitative PCR (qPCR). H3K9me2 levels were determined using primers that amplify from *ura4^+^* to internal loci up to 93 kb from the I-SceI site (Fig. 10A, red bars). Cells containing the uncut 48-bp prototelomere had a localized peak of H3K9me2 near the insertion site, while the fully formed telomere showed a large increase of H3K9me2 spreading (Fig. 10A and D). Spreading of the H3K9me2 mark was under nutritional control, as more spreading was observed in cells grown in rich medium than in cells grown in synthetic medium, even though telomere sizes were nearly identical under both conditions (Fig. 10B). Therefore, similar to changes in Drosophila position effect variegation that respond to temperature (63) and the reversible silencing of S. pombe subtelomere-adjacent genes that are expressed in sporulation medium (25), heterochromatin domains in S. pombe also respond to environmental conditions. In cells with the 0-bp prototelomere and no I-SceI gene, no such enrichment of H3K9me2 was found (Fig. 10C).

**FIG 11:**
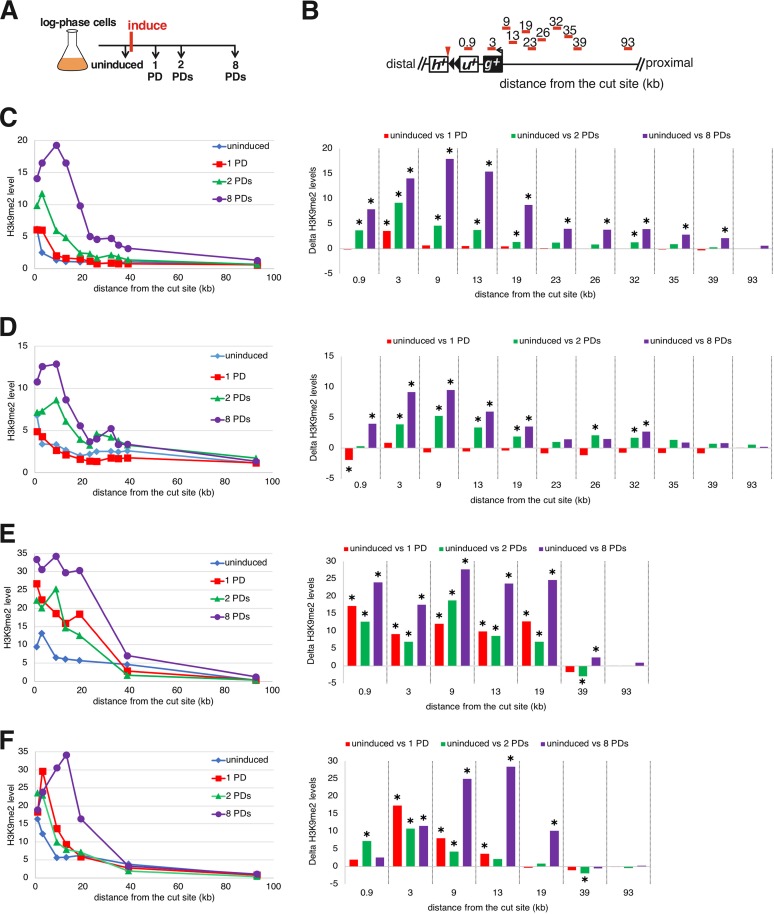
The new H3K9me2 domain forms gradually during new telomere elongation. (A) Telomere formation was induced, and samples were taken at different time points for analysis of telomere lengths and H3K9me2 levels. (B) The primer sets used to monitor the levels of histone H3K9me2 enrichment at several loci are shown as red bars. Distances are relative to the I-SceI cut site, represented as a red arrowhead. The 0.9- and 3-kb probes are in *ura4^+^* (*u^+^*) and *gal1^+^* (*g^+^*), respectively; *h^+^*, *hph^+^*. (C to F) (Right) Four independent kinetic analyses of the levels of histone H3K9me2 enrichment at several loci over ∼8 PDs, normalized to total histone H3 levels at each locus (see Materials and Methods). (Left) Statistical comparisons of ChIP time courses of H3K9me2 spreading compared to uninduced H3K9me2 levels. Statistically significant differences (*P* < 0.05; *t* test) are indicated by asterisks. Statistical method details are provided in “Statistical analysis of ChIP” (Materials and Methods). (The primers for the loci at 23, 36, 32, and 35 kb were omitted in panels D and E.)

**FIG 12:**
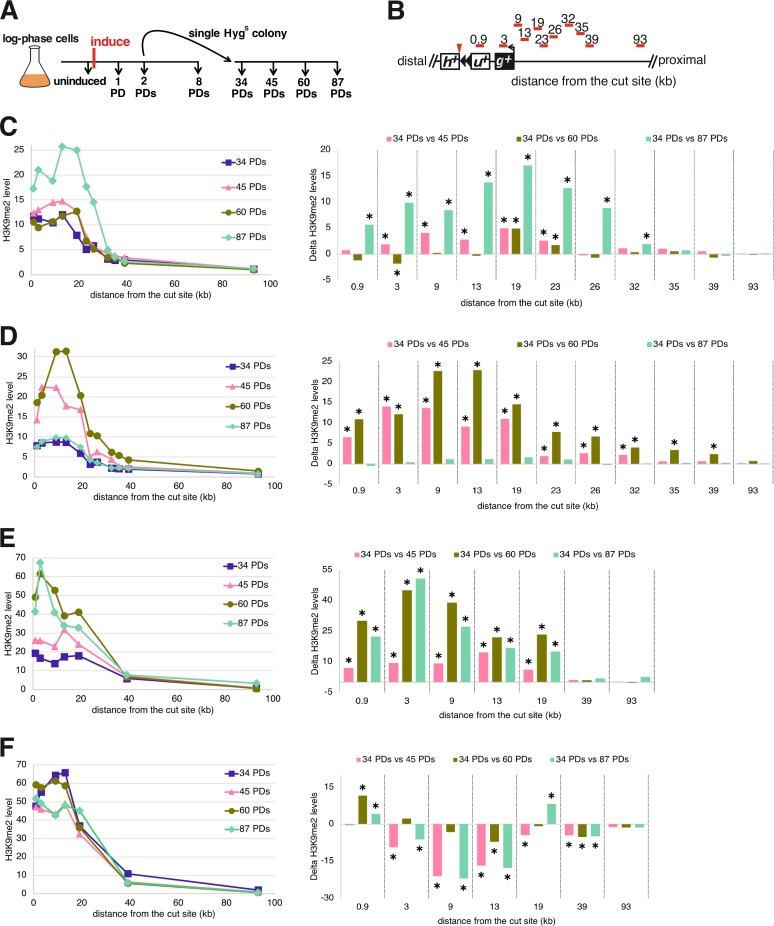
The H3K9me2 domain remains dynamic as the new telomere reaches its equilibrium length. (A) Telomere formation was induced, and samples were taken at different time points for analysis of telomere lengths and H3K9me2 levels. (B) The primer sets used to monitor the levels of histone H3K9me2 enrichment at several loci are shown as red bars. Distances are relative to the I-SceI cut site, represented as a red arrowhead. The 0.9- and 3-kb probes are in *ura4^+^* (*u^+^*) and *gal1^+^* (*g^+^*), respectively; *h^+^*, *hph^+^*. (C to F) (Right) Four independent kinetic analyses of heterochromatin spreading of a single hygromycin-sensitive colony (A), derived from PD 2 of the time courses shown in Fig. 11. (Left) Statistical comparisons of ChIP time courses of H3K9me2 spreading compared to 34-PD H3K9me2 levels. Statistically significant differences (*P* < 0.05; *t* test) are indicated by asterisks, as in Fig. 11.

To understand the relationship between the formation of a functional telomere and the establishment of the telomeric heterochromatin domain, we performed a kinetic analysis of H3K9me2 levels while the new telomere was forming. Upon induction of telomere formation, heterochromatin spreading was monitored in cells grown continuously for 8 PDs (Fig. 11A). To completely eliminate cells where the I-SceI cut is rejoined and healed to cause retention of the *hph^+^* fragment and subtelomere (Fig. 4D), cells from PD 2 (∼8 h of growth) were used to isolate single colonies that were screened for loss of the distal *hph^+^* fragment and then subsequently cultured and analyzed (Fig. 12A).

**FIG 13:**
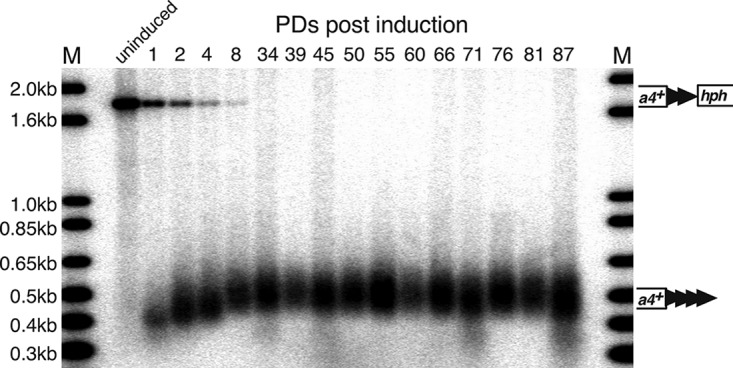
Telomere sizes from a representative experiment from Fig. 11 and 12 were measured at different time points after induction by Southern analysis using a *ura4^+^* probe, as in Fig. 3A. Molecular size standards are shown (lanes M).

In four independent experiments, the H3K9me2 level gradually increased by 0 to 8 PDs and peaked 9 to 20 kb from the cut site (Fig. 11), and telomeres were elongated gradually (Fig. 13). At the 1-PD time point, the cells had short functional telomeres, as the *ura4^+^* telomeric fragment was stable and slightly elongated (Fig. 13), but the H3K9me2 level barely increased (Fig. 11). At 2 and 8 PDs, the size of the heterochromatin slowly increased toward the centromere (Fig. 11), and telomere length reached its equilibrium state at 8 PDs (Fig. 13).

Surprisingly, from PDs 34 to 87, independently formed telomeres from four similar induction assays showed differences in the amounts of heterochromatin at different times in the presence of constant telomere length. These experiments showed spreading of the level of H3K9me2 to a domain of similar size (Fig. 12), and H3K9me2 levels were elevated at the most internal loci at all time points. However, one line (Fig. 12C) showed its highest peak of heterochromatin at 19 kb at 87 PDs. A second line showed an increase at 60 PDs that fell at 87 PDs (Fig. 12D). A third line showed increased levels at 60 PDs and remained high at 87 PDs (Fig. 12E), while the last line showed high levels at all time points (Fig. 12F). Southern analysis revealed that telomere lengths in different formation experiments were indistinguishable at all of these time points (Fig. 10E and 13). Therefore, spreading of the telomeric H3K9me2 mark was dynamic, even though the telomere maintained a constant repeat tract size during this time.

### The *ade6^+^* colony color marker reveals the initiation, spreading, and dynamics of telomeric heterochromatin.

While the slow spreading of heterochromatin and its dynamics at formed telomeres were consistent in each time course assayed as described above, it was noted that the levels of ChIP signal in two time courses performed later in the project (Fig. 11E and F and 12E and F) were about twice the magnitude of those performed 2 years earlier (Fig. 11C and D and 12C and D) by a different researcher. Such uniform changes in ChIP signal levels are frequent in the field when the same assays are performed 2 or more years apart. Examples include the 2-fold differences at the HOOD4 and HOOD10 loci (64, 65) and 2-fold (66, 67) and 3-fold (68, 69) differences at S. pombe telomeric loci. Consequently, the ChIP results shown in Fig. 11 and 12 demonstrate the slow spreading of heterochromatin immediately after telomere formation and the dynamic spreading at established telomeres with a level of variation consistent with results from other groups in the field. However, as the variation led to a level of uncertainty while this work was in review, a ChIP-independent method to analyze the slow spreading and dynamics of heterochromatic gene silencing was also used.

**FIG 14:**
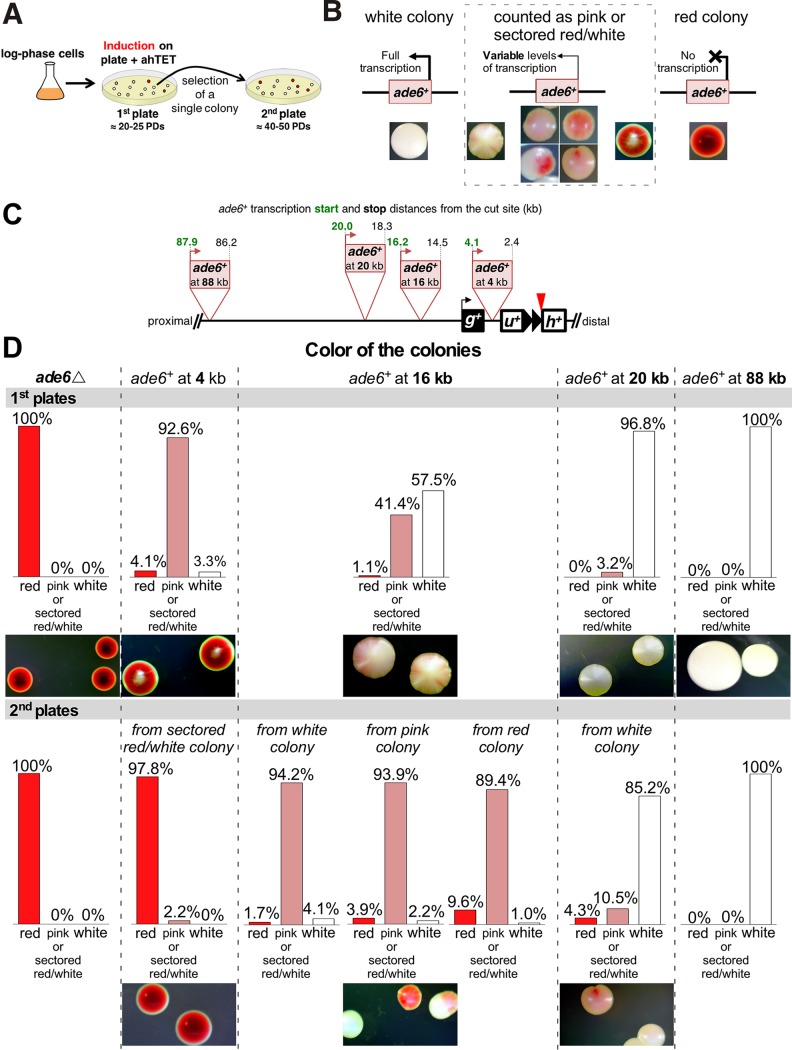
A colony color assay reveals the spreading and dynamic nature of the new heterochromatin domain. (A) Experimental design. Cells were grown for I-SceI induction as for ChIP and induced by plating on plates containing 9 μM ahTET and 7 μg/ml adenine (1st plate). After ∼4 days at 30°C, a single colony from the 1st plate was spread onto a 2nd plate of the same medium. (B) Colony color phenotypes used to classify different levels of *ade6^+^* expression. Full expression forms a white colony, partial or reduced expression results in a pink colony, and loss of expression produces a red colony. Variable or dynamic changes in expression give rise to colonies with different colors, which we grouped into pink or sectored red/white for quantitation. (C) Diagram for the placement and orientation of the different *ade6*^+^ reporters. Distances from the I-SceI cut site and the *ade6*^+^ start codon (in green) and stop codon (in black) are shown. The uninduced strains with these markers form white colonies on the low-adenine medium in these tests. (D) Colony color phenotypes of the first and second platings of the different *ade6^+^* reporters. The dotted lines group the graphs and colony pictures for each *ade6^+^* reporter. The bar graphs show the frequencies of the three types of colonies shown in panel B. At least 1,000 colonies were counted for each plating. Representative colonies are shown, with additional colonies shown in Fig. 15.

The parallel, ChIP-independent analysis of heterochromatin spreading and dynamics used a series of *ade6^+^* genes placed at different distances from the telomere formation site. When grown on medium with limiting amounts of adenine, *ade6^+^* S. pombe cells form white cells and colonies, while cells lacking *ade6^+^* expression and starved for adenine form red cells and colonies. Where *ade6^+^* is repressed near heterochromatin, *ade6^+^* expression can switch between expressed (white cells), partially repressed (pink cells), and repressed (red cells) (21, 70). Cells lacking *ade6^+^* (*ade6*Δ cells) or with the *ade6^+^* reporters integrated at 4 different distances from the I-SceI site were grown as for the ChIP experiments, except that I-SceI expression was induced by plating the cells on medium containing ahTET and small amounts of adenine (Fig. 14A). Induction of telomere formation initiates the spreading of heterochromatin that, based on the above-mentioned H3K9me2 ChIP results (Fig. 11 and 12), requires different numbers of PDs to fully spread. Slow heterochromatin spreading should silence *ade6^+^* genes further from the new telomere at later times during colony growth. As a test of the dynamic extension and retraction of the heterochromatin domain, colonies with different color phenotypes were then picked and plated for single colonies to determine if the repression phenotype of *ade6^+^* changes, as would be predicted if the heterochromatin domain extended or retracted in later PDs (e.g., Fig. 12).

**FIG 15:**
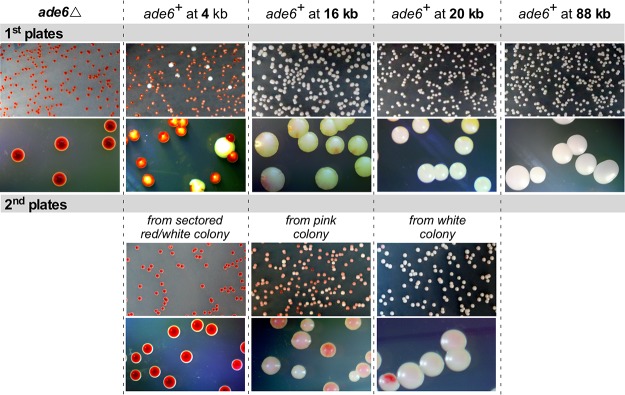
Additional examples of the silencing spreading shown in Fig. 14. The colonies in the different *ade6^+^* integration strains are shown, as in Fig. 14C, as well as the 1st and 2nd plates, as in Fig. 14A.

Control cells bearing only the *ade6*Δ mutation formed uniformly red colonies on the first and second platings of individual cells, as expected for lack of the Ade6 protein (Fig. 14B, C, and D). Cells with the *ade6^+^* start codon 4 kb from the telomere formation site formed colonies with a white center embedded in a dark-red colony (Fig. 14C and D and 15), indicating the presence of the Ade6 protein in early PDs followed by lack of Ade6 protein in later PDs. When colonies with a white center were plated again, almost all of the colonies were dark red like the *ade6*Δ cells (Fig. 14D and 15), similar to the complete repression of the telomeric *ura4^+^* gene in cells that had been propagated with the formed telomere (Fig. 8).

The *ade6^+^* genes with start codons 16, 20, and 88 kb from the telomere formation site also gave colony-sectoring patterns consistent with the H3K9me2 ChIP results. The reporter at 16 kb gave rise to colonies with white centers and pink or red sectors on the outer edges of the colony in the first plating (41%), white colonies where *ade6^+^* had not yet been repressed (58%), and rare red colonies where *ade6*^+^ was repressed (Fig. 14D). However, replating different types of colonies gave rise to the same proportions of colony color phenotypes, where ∼90% of the colonies were pink or red/white sectored with some level of *ade6^+^* repression evident in the center of the colony (Fig. 14D, 2nd plates), consistent with expansion and contraction of the new heterochromatin domain. Some of these red sectored colonies had outer pink sector cells, indicating that repressed *ade6^+^* genes had increased expression during growth, consistent with a retraction of the heterochromatin domain toward the telomere (Fig. 14D and 15, 2nd plates). The *ade6^+^* gene at 20 kb required even more PDs to manifest repression, as 97% of the colonies were white in the first plating and no red colonies were observed. Replating single cells from a white colony gave rise to both red (4%) and pink or red/white sectored (11%) colonies that showed *ade6^+^* repression in the center of the colony. Finally, the *ade6^+^* gene at 88 kb showed no evidence of gene silencing in either the first or second platings of single colonies, consistent with the very low level of H3K9me2 spreading to this locus (Fig. 11 and 12). Thus, the ChIP and *ade6*^+^ expression analyses indicated slow spreading of heterochromatin to form a dynamic gene-silencing domain.

## DISCUSSION

We constructed the first inducible telomere formation system in S. pombe and used it to show that telomeric regions have unexpected properties in healing DSBs and in the kinetics of heterochromatin domain formation and spreading. While inducing a DSB near the middle of the chromosome arm caused significant growth inhibition, DSBs in the subtelomeric region at the 0-bp or 48-bp prototelomere did not (Fig. 3D and E). The 0-bp prototelomere lacking any telomere repeats showed DNA degradation on both sides of the DSB (Fig. 3B) and revealed a backup mechanism to restore telomere function by recombination between a family of subtelomeric repetitive elements (Fig. 6). In contrast, the 48-bp prototelomere with telomere repeats on the centromeric side of the DSB was stable and a substrate for telomere repeat addition, behavior identical to that of a functional short telomere (33, 52, 71) (Fig. 3A, 4, 5, 9, and 13). Formation of a functional telomere was rapid and independent of heterochromatin (Fig. 9); however, establishing the telomere-dependent heterochromatin domain was much slower. The H3K9me2 domain spread gradually from 0 to 8 PDs, when telomere length reached its equilibrium state. The slow spreading of heterochromatin is consistent with the facts that the essential telomere functions in chromosome stability are independent of heterochromatin (22, 57) and that the heterochromatin domain is a secondary consequence of telomere formation. The slow spread of the heterochromatin domain raises the possibility that chromatin domain formation in other biological contexts (e.g., metazoan development, tumorigenesis, and senescence) also requires several cell divisions. After the newly formed telomere repeat tracts reached their final, stable lengths, the size of the H3K9me2 domain remained dynamic, indicating that these changes in the extent of spreading are independent of telomere length.

The backup mechanism to rescue telomere function in response to a subtelomeric DSB reflects the similarities between the genome structures of S. pombe and metazoans. The three nuclear chromosomes of S. pombe have complex subtelomeric regions (51), with the *DUF999* repeats oriented in a way that allowed recombination to attach or copy a functional telomere to the broken chromosome end (Fig. 6). Mammalian genomes also contain a large number of repeats, and small deletions at the border between telomeric euchromatin and heterochromatin are unlikely to cause a phenotype in diploid cells, in contrast to large telomeric deletions, which have developmental consequences (71–73). The S. pombe results therefore suggest that a direct examination of induced DSBs near the border of the mammalian heterochromatic subtelomere may reveal a similar mechanism for rescuing telomere function.

The newly formed S. pombe telomere revealed an unusual heterochromatin domain compared to the uncleaved 48-bp prototelomeres. The uncleaved 48-bp prototelomere showed only a peak of H3K9me2 levels that were centered at the 48-bp telomere repeats (Fig. 10 and 11, uninduced), consistent with the internal telomeric repeats initiating low levels of silencing (22, 23). In contrast, the established telomere with a functional chromosome end formed an internal H3K9me2 heterochromatin domain that peaked from 9 to 26 kb from the telomere (Fig. 12). The reason for this internal peak, as opposed to a peak immediately adjacent to the telomere repeats, is unknown. The location of this internal peak was different in cells grown in rich medium versus synthetic medium (Fig. 10A), suggesting that the peak location is not completely sequence dependent. This S. pombe telomere formation system will thus provide a useful tool for future studies to examine the *cis*- and *trans*-acting factors that regulate the positioning of the H3K9me2 peak.

The telomere formation system revealed a slow and dynamic spreading of the telomeric heterochromatin domain that was not predicted by previous studies. In recent work, where a synthetic S. pombe heterochromatin domain was established by conditionally tethering the H3K9 methyltransferase to an expressed gene, release of the tethered methyltransferase caused the H3K9me2 mark to be lost a few hours later (∼1 or 2 PDs) (12, 13), much faster than the 8 PDs (32 h) required to form the internal H3K9me2 peak (Fig. 11). Assembly of a transcriptionally silenced chromatin in S. cerevisiae, which does not involve H3K9me2, has been monitored, and it also forms at a much higher rate. Overexpression of a silencing protein unique to S. cerevisiae, Sir3, in wild-type cells extended existing Sir3-containing chromatin domains (30–32). An independent approach that used chemical inhibition of silencing and followed its establishment after the inhibitor was withdrawn (74) showed that Sir3 silent chromatin was significantly extended or reestablished in these studies within ∼4 h (∼1 or 2 PDs). Complete modification of the histones as a consequence of Sir3 spreading, however, did require additional PDs (30). In contrast to these events in yeasts, formation of a synthetic heterochromatin domain in murine cells from a tethered silencing factor took much longer (∼5 days) to form the steady-state 10-kb heterochromatin domain (75). While the slower kinetics could be a consequence of the murine tethering system, the S. pombe telomere formation results suggest that the assembly of H3K9me2-dependent heterochromatin domains is an intrinsically slower process than its disassembly.

**FIG 16:**
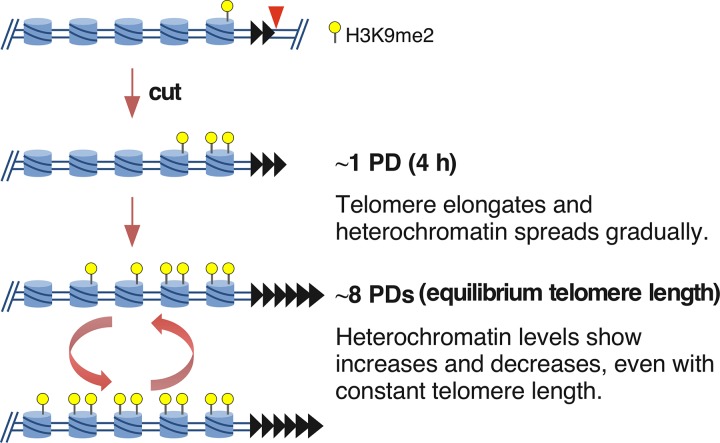
Hypothesis for heterochromatin formation over multiple cell divisions. The 48-bp prototelomere (black triangles) has a low level of H3K9me2 in nucleosomes (blue cylinders) of the adjacent loci. While telomeres are immediately functional for end protection upon I-SceI cleavage, only a small amount of H3K9me2 mark has been established by 1 PD. Spreading gradually increases over 8 PDs. However, the amount of spreading still varies, as cells continue to grow, even though the telomere repeat tract length is constant. These increases may reflect the fact that more cells have both N termini of histone H3 modified by lysine 9 dimethylation in the histone octamer and more extensive spreading of the heterochromatin domain in some cells, or both.

The telomeric H3K9me2 chromatin domain formed in two distinct phases following telomere formation in a wild-type strain background. The first was the spreading over 8 PDs to form the domain where H3K9me2 peaks near 9 kb from the telomere (Fig. 11), even though a substantial fraction of the telomeres were already normal length by 2 PDs (Fig. 13). This mechanism is consistent with current models of spreading (24, 40), in which the extension of the H3K9me2 modification from its nucleation site (i.e., the newly formed telomere) can occur only in S-phase after DNA replication, when new chromatin is formed. If extension occurs only at the border of the H3K9me2 domain and H3K9 methylation is slow, then S-phase exit may limit the extent of heterochromatin extension for that cycle. A second phase of chromatin dynamics occurs during continued propagation of cells with fully elongated telomeres, where the internal peak of H3K9me2 chromatin from 9 to 26 kb can significantly increase or decrease. These ChIP results were confirmed by the variegated expression of the *ade6^+^* genes at 16 and 20 kb from the telomere (Fig. 14 and 15). How these changes occur is unknown and may reflect methylation of the Lys9 residues on both H3 amino termini in the histone octamer. The internal peak of H3K9me2 chromatin may be indicative of a cryptic enhancer of H3K9me2 modification that is activated by this modification spreading from the telomere. In these hypotheses, the changes in H3K9me2 levels occur slowly in continuously growing cultures, consistent with the assembly of new chromatin every new S phase (Fig. 16), although the active replacement of nucleosomes by chromatin-remodeling factors outside S phase cannot be ruled out.

Recently, Obersriebnig et al. (14) examined the spreading of gene silencing and the H3K9me2 modification in the S. pombe silent-mating-type region after reintroduction of the *clr4^+^* gene into a *clr4*Δ cell by mating. Silencing at different distances from the initiation site was monitored by following the loss of expression of fluorescent-protein genes for the first several generations, and H3K9me2 spreading was followed ∼30 divisions later by ChIP (14). Numerical modeling of the rates of spreading, assuming that fluorescent-protein-gene silencing was due to H3K9me2 and heterochromatin spreading, could be divided into global and local effects that produced outcomes similar to the experimental observations. Spreading in the first few cell divisions was consistent with linear spreading from the initiator in the silent-mating-type region (the *cenH* repeat), similar to spreading of the H3K9me2 mark and gene silencing from our newly formed telomere. Despite the similarities of the two systems in these initial stages of spreading, it is important to note that the silent-mating-type region is a highly specialized and well-studied structure containing initiators, enhancers, and boundary elements that both promote and confine heterochromatin to a defined region to permanently extinguish gene expression as an important part of the fission yeast life cycle (76–78). The newly formed telomere does not contain any known elements of this type and may be more similar to the heterochromatin domain formation that occurs during development or senescence, which encompasses genes that are expressed in different cell types (5–7, 25). Additional work on the newly formed telomeric H3K9me2 domain will therefore be required before a detailed comparison with the silent-mating-type region can be made.

The slow extension of the H3K9me2 modification from its nucleation site (e.g., the newly formed telomere) has functional consequences for the formation of larger chromatin domains, which may require many cell cycles. The rate of transition from one cellular state to another during development or aging would be lowered by formation of a chromatin domain. The rate may well be increased early in development, as oocytes have large amounts of maternally deposited histones and histone-modifying enzymes (79, 80), and the increased levels of chromatin components and modifying enzymes could increase the kinetics of chromatin domain formation. In somatic cells, where the modifiers may be at lower levels, the kinetics of domain formation would be slower and might impede aging and tumorigenesis. The S. pombe telomere formation system will provide an ideal model for testing these ideas and identifying the rate-limiting components in chromatin domain formation, with broader implications for metazoans.

## MATERIALS AND METHODS

### Strains and media.

All the S. pombe strains used in this study are shown in Table 1. Selection for strains containing telomere cassettes was performed in Edinburgh minimal medium with sodium glutamate substituted for ammonium chloride (EMMG) without uracil and with appropriate amino acid supplements and 100 μg/ml HygroGold (InvivoGen) (81). Nonselective growth of strains bearing the telomere cassettes was done in EMMG with adenine, histidine, uracil, leucine, lysine, and arginine (EMMG plus AHULKR) and without hygromycin. Preparation of 10 mM ahTET stock and plates was performed as described previously (34). 5-Fluoroorotic acid (5-FOA) plates were yeast nitrogen base plates with 1 mg/ml 5-FOA (Toronto Research Chemicals, Inc.) (http://www-bcf.usc.edu/~forsburg/drugs.html) and with the appropriate supplements. All recombinant DNA procedures were carried out in NEB 5-alpha (New England BioLabs) and TOP10 (Life Technologies) competent cells.

**TABLE 1:** Strains used in this study

Strain	Genotype	Notes
YSLS793	*h*^−^ *leu1-32*::*pDUAL-TETp*-I-PpoI rDNA^I-PpoImt^ *lys1*::I-PpoI^CS^-*hph^+^*	Reference 34
YSLS792	*h*^−^ *leu1-32*::*pDUAL-TETp*-I-PpoI rDNA^I-PpoImt^ *lys1*::*HO^CS^*-*hph^+^*	Reference 34
YJRE49	*h*^−^ *ade6-210 arg3-D4 his3-D1 leu1-32 ura4-D18 hhf1*::*arg3^+^ hhf2*::*his3^+^ hhf3*::*nat*^R^	
YJRE107	*h*^−^ *ade6-210 arg3-D4 his3-D1 leu1-32 ura4-D18 hhf1*::*arg3^+^ hhf2*::*his3^+^ hhf3*::*nat*^R^ *leu1-32*::*pFA-LEU2-TETp*-I-SceI	I-SceI enzyme + no cleavage site
YJRE112	*h*^−^ *ade6-210 arg3-D4 his3-D1 leu1-32 ura4-D18 hhf1*::*arg3^+^ hhf2*::*his3^+^ hhf3*::*nat*^R^ *leu1-32*::*pFA-LEU2-TETp*-I-SceI *gal1-3*′::*ura4^+^* 48-nt *TeloRpt*-I-SceI-*hyg*^R^	I-SceI enzyme + I-SceI site at 48-bp prototelomere
YJRE116	*h*^−^ *ade6-210 arg3-D4 his3-D1 leu1-32 ura4-D18 hhf1*::*arg3^+^ hhf2*::*his3^+^ hhf3*::*nat*^R^ *leu1-32*::*pFA-LEU2-TETp*-I-SceI *gal1-3*′::*ura4^+^* 0-nt *TeloRpt*-I-SceI-*hyg*^R^	I-SceI enzyme + I-SceI site at 0-bp prototelomere
YJRE140	*h*^−^ *ade6-210 arg3-D4 his3-D1 leu1-32 ura4-D18 hhf1*::*arg3^+^ hhf2*::*his3^+^ hhf3*::*nat*^R^ *leu1-32*::*pDUAL-TETp gal1-3*′::*ura4^+^* 48-nt *TeloRpt*-I-SceI-*hyg*^R^	No enzyme + I-SceI site at 48-bp prototelomere
YJRE151	*h*^−^ *ade6-210 arg3-D4 his3-D1 leu1-32 ura4-D18 hhf1*::*arg3^+^ hhf2*::*his3^+^ hhf3*::*nat*^R^ *leu1-32*::*pDUAL-TETp gal1-3*′::*ura4^+^* 0-nt *TeloRpt*-I-SceI-*hyg*^R^	No enzyme + I-SceI site at 0-bp prototelomere
YJRE179	*h*^−^ *ade6-210 arg3-D4 his3-D1 leu1-32 ura4-D18 hhf1*::*arg3^+^ hhf2*::*his3^+^ hhf3*::*nat*^R^ *lys1*::I-SceI *hyg*^R^ *leu1-32*::*pFA-LEU2-TETp*-I-SceI	I-SceI enzyme + I-SceI site at *lys1^+^*
YJRE180	*h*^−^ *ade6-210 arg3-D4 his3-D1 leu1-32 ura4-D18 hhf1*::*arg3^+^ hhf2*::*his3^+^ hhf3*::*nat*^R^ *lys1*::I-SceI *hyg*^R^ *leu1-32*::*pDUAL-TETp*	No enzyme + I-SceI site at *lys1^+^*
YJRE181	*h*^−^ *ade6-210 arg3-D4 his3-D1 leu1-32 ura4-D18 hhf1*::*arg3^+^ hhf2*::*his3^+^ hhf3*::*nat*^R^ *leu1-32*::*pFA-LEU2-TETp*-I-SceI *SPBPB2B2.07c* repaired	0-bp survivor derived from YJRE116
YJRE182	*h*^−^ *ade6-210 arg3-D4 his3-D1 leu1-32 ura4-D18 hhf1*::*arg3^+^ hhf2*::*his3^+^ hhf3*::*nat*^R^ *leu1-32*::*pFA-LEU2-TETp*-I-SceI *gal1-3*′::*ura4^+^* 48^+++^-nt *TeloRpt*	48-bp form derived from YJRE112
YJRE210	*h^+^ ade6-210 arg3-D4 his3-D1 leu1-32 ura4-D18 leu1-32*::*pFA-LEU2-TETp*-I-SceI *gal1-3*′::*ura4^+^* 48-nt *TeloRpt*-I-SceI-*hyg*^R^	I-SceI enzyme + I-SceI site at 48-bp prototelomere
YJRE212	*h^+^ ade6-210 arg3-D4 his3-D1 leu1-32 ura4-D18 leu1-32*::*pFA-LEU2-TETp*-I-SceI *gal1-3*′::*ura4^+^* 0-nt *TeloRpt*-I-SceI-*hyg*^R^	I-SceI enzyme + I-SceI site at 0-bp prototelomere
JA007-1	*h^+^ clr4*::*kanMX6 ade6-210 arg3-D4 his3-D1 leu1-32 ura4-D18 leu1-32*::*pFA-LEU2-TETp*-I-SceI *gal1-3*′::*ura4^+^* 48-nt *TeloRpt*-I-SceI-*hyg*^R^	*clr4*Δ I-SceI enzyme + I-SceI site at 48-bp prototelomere
JA011-1	*h^+^ TER1*::*kanMX6 ade6-210 arg3-D4 his3-D1 leu1-32 ura4-D18 leu1-32*::*pFA-LEU2-TETp*-I-SceI *gal1-3*′::*ura4^+^* 48-nt *TeloRpt*-I-SceI-*hyg*^R^	TER1Δ I-SceI enzyme + I-SceI site at 48-bp prototelomere
JA018-2	*chk1-13myc*::*kanMX6 ade6-210 arg3-D4 his3-D1 leu1-32 ura4-D18 leu1-32*::*pFA-LEU2-TETp*-I-SceI *gal1-3*′::*ura4^+^* 48-nt *TeloRpt*-I-SceI-*hyg*^R^	Chk1-myc in I-SceI enzyme + I-SceI site at 48-bp prototelomere
JA046-1	*chk1-13myc*::*kanMX6 ade6-210 arg3-D4 his3-D1 leu1-32 ura4-D18 leu1-32*::*pFA-LEU2-TETp*-I-SceI *gal1-3*′::*ura4^+^* 0-nt *TeloRpt*-I-SceI-*hyg*^R^	Chk1-myc in I-SceI enzyme + I-SceI site at 0-bp prototelomere
JA057-1	*h^+^ ade6*::*kanMX6 ade6-210 arg3-D4 his3-D1 leu1-32 ura4-D18 leu1-32*::*pFA-LEU2-TETp*-I-SceI *gal1-3*′::*ura4^+^* 48-nt *TeloRpt*-I-SceI-*hyg*^R^	*ade6*Δ I-SceI enzyme + I-SceI site at 48-bp prototelomere
JA058-1	*chk1-13myc*::*kanMX6 ade6-210 arg3-D4 his3-D1 leu1-32 ura4-D18 hhf1*::*arg3^+^ hhf2*::*his3^+^ hhf3*::*nat*^R^ *lys1*::I-SceI *hyg*^R^ *leu1-32*::*pFA-LEU2-TETp*-I-SceI	Chk1-myc in I-SceI enzyme + I-SceI site at *lys1^+^*
JA064-1	*h+ ade6*::*kanMX6 ade6-210 arg3-D4 his3-D1 leu1-32 ura4-D18 leu1-32*::*pFA-LEU2-TETp*-I-SceI *gal1-3*′::*ura4^+^* 48-nt *TeloRpt*-I-SceI-*hyg*^R^ *ade6^+^* (at kb 20 downstream I-SceI site)	*ade6^+^* at kb 20 to I-SceI site (48-bp prototelomere + I-SceI enzyme)
JA066-1	*h^+^ ade6*::*kanMX6 ade6-210 arg3-D4 his3-D1 leu1-32 ura4-D18 leu1-32*::*pFA-LEU2-TETp*-I-SceI *gal1-3*′::*ura4^+^* 48-nt *TeloRpt*-I-SceI-*hyg*^R^ *ade6^+^* (at kb 88 downstream I-SceI site)	*ade6^+^* at kb 88 to I-SceI site (48-bp prototelomere + I-SceI enzyme)
JA067-1	*h^+^ ade6*::*kanMX6 ade6-210 arg3-D4 his3-D1 leu1-32 ura4-D18 leu1-32*::*pFA-LEU2-TETp*-I-SceI *gal1-3*′::*ura4^+^* 48-nt *TeloRpt*-I-SceI-*hyg*^R^ *ade6^+^* (at kb 4 downstream I-SceI site)	*ade6^+^* at kb 4 to I-SceI site (48-bp prototelomere + I-SceI enzyme)
JA068-1	*h^+^ ade6*::*kanMX6 ade6-210 arg3-D4 his3-D1 leu1-32 ura4-D18 leu1-32*::*pFA-LEU2-TETp*-I-SceI *gal1-3*′::*ura4^+^* 48-nt *TeloRpt*-I-SceI-*hyg*^R^ *ade6^+^* (at kb 16 downstream I-SceI site)	*ade6^+^* at kb 16 to I-SceI site (48-bp prototelomere + I-SceI enzyme)

### I-SceI expression vector.

I-SceI is produced from a synthetic gene with optimized S. pombe codons (47) and expressed as a protein with two N-terminal simian virus 40 (SV40) NLS fused to I-SceI. I-SceI expression was under the cauliflower mosaic virus 35S promoter (CaMV35Sp), which is regulated by the tetracycline repressor (TetR). The TetR protein is produced from the *adh1^+^* promoter in the same cassette as I-SceI (83). pFA-LEU2-I-SceI was produced by a 5-part recombination cloning in S. cerevisiae, rescued to bacteria, and verified by DNA sequencing (84). An I-SceI site on the vector backbone was removed by site-directed mutagenesis. Additional cloning details are available upon request.

### Telomere cassette.

The last unique sequence of S. pombe chromosome IIR was found to be the 2-kb region 3′ of the *gal1^+^* gene 3′ untranslated region (UTR) (44). The prototelomere cassette containing *ura4^+^*, 0 or 48 bp of telomere seeding sequence, and the *hph^+^* gene encoding hygromycin resistance was constructed in the vector pRS315 by 5-part recombination cloning in S. cerevisiae. The junctions between DNA fragments were verified by colony PCR, and the plasmids were rescued to bacteria and sequenced. Additional cloning details are available upon request. The vector and its sequence have been deposited with Addgene.

### Construction of the I-SceI *lys1^+^* allele.

The I-PpoI site in the plasmid pSS23 (34) was replaced by the I-SceI site by standard cloning. Transformation, selection for the hygromycin resistance gene *hph^+^*, and confirmation of integration of I-SceI at *lys1^+^* in S. pombe was done as described previously (34).

### Induction of I-SceI.

Cells containing the telomere cassettes were grown under selection overnight, diluted to a final volume of 230 ml at 5.5 × 10^6^ cells/ml in nonselective medium, and grown for 3.75 h. Untreated cells (3 × 10^8^ to 5 × 10^8^) were removed and pelleted, washed once with sterile water, and frozen at −80°C. Anhydrotetracycline was then added to a final concentration of 9 μM. Cells then were collected at various time points and pelleted, washed, and frozen as described above. Genomic DNA was extracted (85) from the frozen pellets for Southern analysis as discussed below. I-SceI cleavage at *lys1^+^* was performed and analyzed as described previously (34), except that ahTET was added at a final concentration of 9 μM.

### Southern blot analysis.

Cells (3 × 10^8^ to 5 × 10^8^) were collected at each time point and used to prepare genomic DNA (85). The genomic DNA (5 μg) was digested with 20 units of ScaI and analyzed via Southern blotting with α-^32^P-labeled probes produced by PCR with u4ScaProbe_S plus u4ScaProbe_AS (*ura4^+^* probe) or SV40ScaProbe_S plus SV40ScaProbe_AS (*hph^+^* probe) or with abo1-probe_S plus abo1-probe_AS (*abo1^+^* probe as a loading control) (Table 2). The purified PCR product (50 ng) was denatured and treated with 10 units of Klenow (New England BioLabs) in the presence of primers (final concentration, 0.25 μM) and 100 μCi of [α-^32^P]dATP or [α-^32^P]dCTP (3,000 to 6,000 Ci/mmol; PerkinElmer) in a 40-μl reaction mixture at 37°C for 30 min. The probe was purified in a G-25 spin column (GE Healthcare Life Sciences), and 2 × 10^6^ to 10 × 10^6^ cpm was used in Southern blot hybridization. Prehybridization and hybridization were performed with PerfectHyb (Sigma) as described previously (86). Stripping of the membrane was performed in buffer containing 0.5% SDS and 0.1× SSC (1× SSC is 0.15 M NaCl plus 0.015 M sodium citrate) with heating to 100°C twice for 15 min each time.

**TABLE 2:** Primers used in this study

Name^*a*^	Sequence	Purpose^*b*^
914 lys1 5′ I-PpoI	TCGAACATTTGACACTCTCCG	1
924 I-PpoI hph 3′	CCACACCCTAACTGACAAGATC	1
920 his3 RT For	TTACCAAGCCACTAACACCAG	1
921 his3 RT Rev	GCAGAGACCGTATACATTCCG	1
u4ScaProbe_S	GTAGCGACTAAAATATTAACTATTATAG	2
u4ScaProbe_AS	AAACTTTTTGACATCTAATTTATTCTG	2
SV40ScaProbe_S	CGGATCTGATCAGCACGTGTTG	2
SV40ScaProbe_AS	TGTCAAGCACTTCCGGAATC	2
abo1-probe_S	CTGCAGCTTGTTCTTCCGAA	2
abo1-probe_AS	GCATCGAACATAGCCATTCA	2
u4-teloPCR-1S	CCAATGAAAGATGTATGTAGATGAATG	3
BamHI-G_18_	CGGGATCCGGGGGGGGGGGGGGGGGG	3
M13R	CAGGAAACAGCTATGAC	3
M13F	TGTAAAACGACGGCCAGT	3
gal1_ChIP_S	[SCAP]CCCGAGTTAAGAGAGGTATG[R]	4
gal1_ChIP_AS	[SCAP]GGTTTCGATACGACAACAGC[R]	4
ura4ChIP_F	CAAGGCCTCAAAGAAGTTGG	4, 5
ura4ChIP_R	GATGATATCGCTACCGCAG	4, 5
SPBPB2B2.06c-ChIP-S (23 kb)	CACAATTATCACTCCATTCTTGTTG	4
SPBPB2B2.06c-ChIP-AS (23 kb)	CCTTCAAAGTATCGTGCTAAAGG	4
SPBPB2B2.09c-ChIP-S (13 kb)	CGAAATAATGTCAATGGACCGAAG	4
SPBPB2B2.09c-ChIP-AS (13 kb)	GTGCCAACAGTTCATCTACCAGG	4
SPBPB2B2.12c-ChIP-S (6 kb)	CGTATGGATTGGTTGGGTCATTAG	4
SPBPB2B2.12c-ChIP-AS (6 kb)	GGGTGTTATTCATTTTGCGGC	4
SPBPB2B2.08-ChIP-S (9 kb)	CTTAAAAGCTGTTCGTGGGTGG	4
SPBPB2B2.08-ChIP-AS (9 kb)	TGGTTCTTATGGTTGGACACTGG	4
SPBPB2B2.07c-ChIP-S (19 kb)	TCTTTTTTCACGAGGGTAATGCTG	4, 5
SPBPB2B2.07c-ChIP-AS (19 kb)	TCTCGCTTTCATCATTGTTTTGC	4, 5
50kb-2-S (39 kb)	GGAGAGCAGCTTTACCTTGTG	4, 6
50kb-2-AS (39 kb)	CGACCAGCACCTCTACCTCT	4, 6
07c-2-AS-rvs&compl	AGCAAGCACGCAAACTCTG	4
07c-08-2-S	TGTCAAACATCCGCATACAA	4
07c-08-2-AS	CGGTCTATTTGTTCCCATGC	4
07c-08-3-S	TGCTGTCGCAAAACAGAAAG	4
07c-08-3-AS	GACGCAAGATTGGTTGGAAT	4
07c-08-5-S	GCCCATCTTCCTTATGCTCA	4
07c-08-5-AS	AAACGTTTGCTTCCAAATGC	4
post-DUF-AS	AAGCTACGCAGTTTGGTATCTG	4
post-LTR-AS	AAGAAAACTCATCGCAGCCTA	4
post-dust-AS	TCCTTGTACGAATCAAAGGAAT	4
DUF-8-AS	ATGAACTGGCTAAGCGGAAC	4
DUF-9-AS	ACTGCGGAACGAAAATAAGG	4
DUF-10-AS	GGAAAAACGGCATTACAGTCA	4
BsrDI-map-AS	ACCTACTCACAACGGGGAAA	4
ura4-3-S (0.9 kb)	TGGCTACTGGTTCCTACACA	6
ura4-3-AS (0.9 kb)	CCCGTCTCCTTTAACATCCA	6
gal1-5-S (3 kb)	GGACAGCGATGATCCTCAAA	6
gal1-5-AS (3 kb)	CTCCATAACCTCCGTTAGCC	6
gal7-2-S (9 kb)	CTCTCACCGCAGGATAGTCA	6
gal7-2-AS (9 kb)	TGGCAGGGAGCTAAAGAAGA	6
09c-2-S (13 kb)	GGGATTCTTTGGGACTTTGG	6
09c-2-AS (13 kb)	GTTTGTGGCAAGTTGTGG	6
07c-2-S (19 kb)	GAGAGCCCAATGACTGAA	6
07c-2-AS (19 kb)	CAGAGTTTGCGTGCTTGCT	6
06c-3-S (23 kb)	GACTGGCGAGTTTCATTGGT	6
06c-3-AS (23 kb)	AATCTTTGGCGGTCACTCTC	6
SPBPB2B2.05-ChIP-S (26 kb)	CTCTGACGACGGACTTTGTG	6
SPBPB2B2.05-ChIP-AS (26 kb)	TGCCACTTTGAGCGGTTT	6
mug180-1-S (32 kb)	GGCTCGCAAAGGTTAATGG	6
mug180-1-AS (32 kb)	GGTGGTCTGTTCATCGTCGT	6
01-2-S (35 kb)	ATGTCGGTGAGGTCGAAACA	6
01-2-AS (35 kb)	CGACGCTTTAGTCCTTGTCC	6
100kb-2-S (93 kb)	GAAGGGCGAGGCACATTAC	6
100kb-2-AS (93 kb)	GGTGATCCCACACACGACT	6
act1 Fw-EOS141	TGCCGATCGTATGCAAAAGG	6
act1 Rv-EOS142	CCGCTCTCATCATACTCTTG	6

a The distance to the cut site for the primers used in Fig. 6, 10, 11, and 12 is noted in parentheses. The primers act1 Fw-EOS141 and Rv-EOS142 were from reference 68.

b The purpose of each primer is indicated as follows: 1, qPCR for I-SceI-*lys1^+^* (93); 2, Southern analysis probe; 3, telomere PCR and sequencing; 4, PCR analysis and mapping of 0-bp survivors; 5, PCR to test for the presence of *ura4^+^*; 6, qPCR of ChIP samples.

### Single-cell analysis of cell growth after I-SceI induction.

Cells were grown in liquid under selection for the prototelomere or I-SceI site cassette overnight at 32°C. The cells were then transferred to 5 ml of nonselective EMMG-based medium at a concentration of 5.5 × 10^5^ cells/ml and allowed to recover for 3.75 h before spotting on a nonselective EMMG-based plate with 9 μM ahTET. The cells were placed ∼4 mm apart on a 4-by-11 grid by micromanipulation and were observed 3, 6, 9, and 25 h after plating. The extent of growth was classified into 6 categories: 1 cell (starting condition), 2 cells, 3 or 4 cells, 5 to 8 cells, 9 to 16 cells, and >16 cells. The plates were incubated for a total of 3 days at 32°C. The plates were scanned, replica plated on nonselective EMMG-based plates and in yeast extract-sucrose (YES)-plus-hygromycin plates, incubated for another 3 days at 32°C, and scanned.

### Telomere PCR and sequencing.

Telomere PCR was performed as previously described (86, 87) with primers u4-teloPCR-1S and BamHI-G_18_ (Table 2) using genomic DNA from 1, 8, and 50 PDs. Cells from 8 PDs (32 h) were spread for single colonies. A portion of each colony was tested for hygromycin sensitivity. Two hygromycin-sensitive colonies were used for this analysis as 50-PD clones 1 and 2. The purified telomere PCR products were cloned into a TOPO vector (Life Technologies) and sequenced using M13F or M13R primers (Table 2) at the Lerner Research Institute Genomics Core.

### ahTET plating assay.

The spot test assay was performed by spotting 5-fold serial dilutions onto the indicated plates as described by Sunder et al. (34). Strains containing prototelomere constructs were grown without ahTET and under selection for the telomere cassette and then plated on nonselective EMMG with and without ahTET. For the quantitative-plating assay, cells were plated onto nonselective EMMG with or without ahTET at 300 cells per plate and grown for 4 to 5 days at 30°C. The average number of colonies from three individual plates with ahTET was normalized to that from plates without ahTET for strains containing the I-SceI gene. This was then normalized to the same ratio of control cells without the I-SceI gene. Statistical comparisons were performed using Prism version 6.0 (GraphPad Software).

### Selection of 0-bp survivors.

S. pombe cells containing the 0-bp prototelomere were induced with ahTET (9 μM final concentration) and grown overnight in liquid EMMG. Cells were struck for single colonies on rich medium and grown for 3 days. The resulting colonies were tested for sensitivity to hygromycin (100 μg/ml). DNA was extracted from 3 separate isolates that were sensitive to hygromycin and analyzed via PCR, using primers listed in Table 2, to determine which sequences had been deleted after initiating the DSB at the 0-bp prototelomere.

### Mapping of 0-bp survivors.

The recombination site was determined using inverse-circle PCR. Briefly, genomic DNA from 3 separate isolates (5 μg) was digested with 20 units of EcoRI for 16 h at 37°C, followed by inactivation at 65°C for 20 min. A portion of the digest (1 μg) was diluted in a ligation reaction to a total volume of 200 μl using 40 units of T4 DNA ligase (New England BioLabs) for 16 h at 18°C. The ligation was ethanol precipitated and resuspended in 10 μl of 10 mM Tris-1 mM EDTA, pH 8.0. Half of the product was amplified with primers 07c-2-AS-rvs&compl and BsrDI-map-AS, and the product was sequenced with the same primers (Table 2). The resulting sequence was subjected to BLAST analysis and aligned with the S. pombe genome (44).

### *ura4*^+^ silencing assay.

Cells containing the telomere cassettes were grown under selection overnight. The cells were then transferred to 5 ml of nonselective medium at a concentration of 5.5 × 10^5^ cells/ml and allowed to recover for 3.75 h before addition of ahTET (9 μM final concentration). Cells (1 × 10^6^) were collected before and after overnight induction with ahTET, and 5 × 10^5^ cells were plated in 5-fold serial dilutions on plates with the media indicated and grown for 3 or 4 days at 30°C.

### *ade6*^+^ silencing assay.

Cells containing the telomere cassettes and the *ade6*Δ mutation and bearing different *ade6^+^* integrations were grown under selection overnight. The cells were then transferred to 5 ml of nonselective medium at a concentration of 5.5 × 10^5^ cells/ml and allowed to recover for 3.75 h before plating for single cells on nonselective medium with 9 μM ahTET and 7 μg/ml adenine. Cells were plated at 10 to 300 per plate to obtain single colonies and incubated for 3 to 4 days at 30°C.

### Analysis of *ura4^+^* in the Ura^−^ 5-FOA-resistant colonies.

Single colonies resistant to 5-FOA (which Ura4 converts to a poison) after induction of I-SceI were tested for hygromycin sensitivity on rich medium. DNA was extracted and analyzed for the presence of *ura4^+^* by PCR using 5 Prime HotMaster *Taq* DNA polymerase (Quantbio) according to the manufacturer's instructions and primers ura4ChIP_F and ura4ChIP_R (Table 2) and an extension time of 1.0 min for 25 cycles (MJ Research PTC 200 thermal cycler). A positive control for all PCRs was performed in parallel using primers SPBPB2B2.07c-ChIP-S and SPBPB2B2.07c-ChIP-AS (Table 2) to amplify the *DUF999-7* gene and produced a product in all the reactions.

### ChIP assay.

At each time point, cells in 300 ml at an optical density at 600 nm (OD_600_) of 0.8 to 1.2 were cross-linked with 1% formaldehyde and washed twice with cold HBS buffer (50 mM HEPES-NaOH, pH 7.5, 140 mM NaCl). The cell pellets were stored at −80°C. For a saturated culture, cells were diluted to the above OD_600_ for cross-linking. At ∼2 PDs (8 h), cells were struck for single colonies on rich medium for 3 to 4 days at 30°C. Portions of several resulting colonies were tested for sensitivity to hygromycin (100 μg/ml). A hygromycin-sensitive colony was inoculated in nonselective EMMG with ahTET, and cells from serial dilutions were collected for analysis. All subsequent steps were performed at 4°C. Cell pellets were resuspended in ChIP lysis buffer (88) and lysed using mechanical disruption with a bead beater (Bio Spec Mini-Beadbeater-16) with 0.5-mm glass beads (Biospec 11079105) using 4 cycles of 45 s followed by 60 s on ice. The lysate was sonicated for 10 cycles on maximum power (30 s on and 59 s off) in a Diagenode Bioruptor XL with sample tubes soaked in an ice water bath. Solubilized chromatin protein (2 to 4 mg) was used for each ChIP, while 5 μl was saved as input. Antibodies (2 μg) against H3K9me2 (Abcam; ab1220) or total H3 (Abcam; ab1791) were added to the lysate and incubated with rocking for 4 h at 4°C. Dynabeads Protein G (50 μl; Life Technologies) was then added to the lysate for rocking overnight at 4°C. The beads were washed with ChIP lysis buffer, ChIP lysis buffer with 500 mM NaCl, wash buffer, and TE buffer (10 mM Tris, 1 mM EDTA, pH 7.5) successively (88). The beads were then resuspended in 145 μl of TES (1× TE with 1% SDS). The supernatant (120 μl) was recovered and incubated in a Thermomixer (Eppendorf) at 65°C, 1,000 rpm overnight to reverse cross-linking. For input samples, TES buffer (115 μl) was added and incubated in the Thermomixer with the ChIP samples. Samples were treated with RNase A and proteinase K and purified on a Qiagen PCR purification column (89). All time points from the same induction assay were processed for ChIP assay at the same time.

### qPCR analysis for ChIP.

Input samples were diluted to 1/100 with double-distilled H_2_O (ddH_2_O), while beads-only ChIP, H3 ChIP, and H3K9me2 ChIP samples were diluted to 1/10. Template DNA (4 μl) was added to 5 μl of Roche LightCycler 480 SYBR green I master (2×), and primers were added to a final concentration of 0.6 μM for a 10-μl total reaction volume. Each sample was run in triplicate on the same 384-well PCR plate (Roche LightCycler 480 Multiwell Plate 384, clear) in a Roche LightCycler 480. Each ChIP assay was performed at least three times independently. H3K9me2 levels were normalized to the total H3 levels at each locus (90–92), and each ratio was normalized to the *act1^+^* control locus in the same ChIP (93). Fold enrichments (FE) were calculated using the ΔΔ*C_q_* method for each locus at each time point for a locus of interest (loi) as follows: ${\text{FE}}_{\text{loi}}=\frac{2^{-[({Cq}_{\text{H}3\text{K}9\text{me}2}\;-\;A)\;-\;({Cq}_{\text{beads}}\;-\;A)]}}{2^{-[({Cq}_{\text{H}3}\;-\;A)\;-\;({Cq}_{\text{beads}}\;-\;A)]}}$, where *A* = *Cq*_Input_ − log_2_ (dilution factor). Then, the FE of the loi was normalized to the FE of *act1^+^* to generate the final FE of H3K9me2 at each locus.

### Statistical analysis of ChIP.

Figures 10, 11, and 12 show independent induction assays in which all time points from each ChIP assay were processed at the same time. ChIP assays and qPCR were performed as described in “ChIP assay” and “qPCR analysis for ChIP” above. Each H3K9me2 level at each locus is the average of three independent qPCRs processed from the same DNA sample (technical repeats). Statistical comparisons were performed using an unpaired *t* test with these technical repeats. The four independent biological replicates shown in Fig. 11 compared H3K9me2 levels in the uninduced sample with those at 1, 2, or 8 PDs within the same time course. The four independent biological replicates in Fig. 12 compared the H3K9me2 levels in the 34-PD sample with those at 45, 60, and 87 PDs within the same time course. Time points with *P* values of less than 0.05 are marked with asterisks in Fig. 11 and 12. ChIP assays in Fig. 10A and C, 11C and D, and 12C and D were performed by J. Wang in 2014 and 2015, and those in Fig. 10D, 11E and F, and 12E and F were performed by J. Audry in 2016 and 2017.

### Western blot analysis.

Whole-cell extracts were prepared from exponentially growing cells. The protein concentration was normalized using the colorimetric Bradford method. Total proteins (25 μg) were then separated by SDS-PAGE on a precast 7.5% gel (Mini-Protean TGX; Bio-Rad) and transferred to a nitrocellulose membrane. Blots were blocked with Odyssey blocking buffer–phosphate-buffered saline (PBS) (Li-Cor). Chk1-myc detection used a mouse anti-myc antibody (9B11; Cell Signaling, 1:1,000 in Odyssey blocking buffer-PBS plus 0.2% Tween 20), followed by a donkey anti-mouse IRDye 680RD secondary antibody (Li-Cor; 1:15,000 in Odyssey blocking buffer-PBS plus 0.2% Tween 20). Blot development used near-infrared (NIR) imaging on an Odyssey CLx instrument (Li-Cor).

## References

[1] Chaligné R, Heard E 2014 X-chromosome inactivation in development and cancer. FEBS Lett 588:2514–2522. doi:10.1016/j.febslet.2014.06.023. 24937141

[2] Lunyak VV, Rosenfeld MG 2008 Epigenetic regulation of stem cell fate. Hum Mol Genet 17:R28–R36. doi:10.1093/hmg/ddn149. 18632693

[3] Sharma S, Kelly TK, Jones PA 2010 Epigenetics in cancer. Carcinogenesis 31:27–36. doi:10.1093/carcin/bgp220. 19752007 PMC2802667

[4] Lodish H, Berk A, Matsudaira P, Kaiser CA, Krieger M, Scott MP, Zipursky SL, Darnell J 2007 Molecular cell biology, 6th ed W. H. Freeman and Company, New York, NY.

[5] Funayama R, Ishikawa F 2007 Cellular senescence and chromatin structure. Chromosoma 116:431–440. doi:10.1007/s00412-007-0115-7. 17579878

[6] Rai TS, Adams PD 2012 Lessons from senescence: chromatin maintenance in non-proliferating cells. Biochim Biophys Acta 1819:322–331. doi:10.1016/j.bbagrm.2011.07.014. 21839870 PMC3895594

[7] Corpet A, Stucki M 2014 Chromatin maintenance and dynamics in senescence: a spotlight on SAHF formation and the epigenome of senescent cells. Chromosoma 123:423–436. doi:10.1007/s00412-014-0469-6. 24861957

[8] Alper BJ, Lowe BR, Partridge JF 2012 Centromeric heterochromatin assembly in fission yeast—balancing transcription, RNA interference and chromatin modification. Chromosome Res 20:521–534. doi:10.1007/s10577-012-9288-x. 22733402 PMC3580186

[9] Ekwall K 2007 Epigenetic control of centromere behavior. Annu Rev Genet 41:63–81. doi:10.1146/annurev.genet.41.110306.130127. 17711387

[10] Pidoux AL, Allshire RC 2005 The role of heterochromatin in centromere function. Philos Trans R Soc Lond B Biol Sci 360:569–579. doi:10.1098/rstb.2004.1611. 15905142 PMC1569473

[11] Pidoux AL, Allshire RC 2004 Kinetochore and heterochromatin domains of the fission yeast centromere. Chromosome Res 12:521–534. doi:10.1023/B:CHRO.0000036586.81775.8b. 15289660

[12] Audergon PNCB, Catania S, Kagansky A, Tong P, Shukla M, Pidoux AL, Allshire RC 2015 Epigenetics. Restricted epigenetic inheritance of H3K9 methylation. Science 348:132–135. doi:10.1126/science.1260638. 25838386 PMC4397586

[13] Ragunathan K, Jih G, Moazed D 2015 Epigenetics. Epigenetic inheritance uncoupled from sequence-specific recruitment. Science 348:1258699. doi:10.1126/science.1258699. 25831549 PMC4385470

[14] Obersriebnig MJ, Pallesen EMH, Sneppen K, Trusina A, Thon G 2016 Nucleation and spreading of a heterochromatic domain in fission yeast. Nat Commun 7:11518. doi:10.1038/ncomms11518. 27167753 PMC4865850

[15] Muller HJ 1938 The remaking of chromosomes. Collect Net 8:181–198.

[16] McClintock B 1941 The stability of broken ends of chromosomes in zea mays. Genetics 26:234–282. 17247004 10.1093/genetics/26.2.234 PMC1209127

[17] Watson JD 1972 Origin of concatemeric T7 DNA. Nat New Biol 239:197–201. 4507727 10.1038/newbio239197a0

[18] Olovnikov AM 1973 A theory of marginotomy. J Theor Biol 41:181–190. doi:10.1016/0022-5193(73)90198-7. 4754905

[19] Smogorzewska A, de Lange T 2004 Regulation of telomerase by telomeric proteins. Annu Rev Biochem 73:177–208. doi:10.1146/annurev.biochem.73.071403.160049. 15189140

[20] Gottschling DE, Aparicio OM, Billington BL, Zakian VA 1990 Position effect at S. cerevisiae telomeres: reversible repression of Pol II transcription. Cell 63:751–762. doi:10.1016/0092-8674(90)90141-Z. 2225075

[21] Nimmo ER, Cranston G, Allshire RC 1994 Telomere-associated chromosome breakage in fission yeast results in variegated expression of adjacent genes. EMBO J 13:3801–3811. 8070408 10.1002/j.1460-2075.1994.tb06691.x PMC395293

[22] Castillo AG, Pidoux AL, Catania S, Durand-Dubief M, Choi ES, Hamilton G, Ekwall K, Allshire RC 2013 Telomeric repeats facilitate CENP-A(Cnp1) incorporation via telomere binding proteins. PLoS One 8:e69673. doi:10.1371/journal.pone.0069673. 23936074 PMC3729655

[23] Kanoh J, Sadaie M, Urano T, Ishikawa F 2005 Telomere binding protein Taz1 establishes Swi6 heterochromatin independently of RNAi at telomeres. Curr Biol 15:1808–1819. doi:10.1016/j.cub.2005.09.041. 16243027

[24] Elgin SCR, Grewal SIS 2003 Heterochromatin: silence is golden. Curr Biol 13:R895–R898. doi:10.1016/j.cub.2003.11.006. 14654010

[25] Hansen KR, Burns G, Mata J, Volpe TA, Martienssen RA, Bähler J, Thon G 2005 Global effects on gene expression in fission yeast by silencing and RNA interference machineries. Mol Cell Biol 25:590–601. doi:10.1128/MCB.25.2.590-601.2005. 15632061 PMC543407

[26] Hall IM, Shankaranarayana GD, Noma K-I, Ayoub N, Cohen A, Grewal SIS 2002 Establishment and maintenance of a heterochromatin domain. Science 297:2232–2237. doi:10.1126/science.1076466. 12215653

[27] Partridge JF, DeBeauchamp JL, Kosinski AM, Ulrich DL, Hadler MJ, Noffsinger VJP 2007 Functional separation of the requirements for establishment and maintenance of centromeric heterochromatin. Mol Cell 26:593–602. doi:10.1016/j.molcel.2007.05.004. 17531816

[28] Sadaie M, Iida T, Urano T, Nakayama J-I 2004 A chromodomain protein, Chp1, is required for the establishment of heterochromatin in fission yeast. EMBO J 23:3825–3835. doi:10.1038/sj.emboj.7600401. 15372076 PMC522800

[29] Schalch T, Job G, Noffsinger VJ, Shanker S, Kuscu C, Joshua-Tor L, Partridge JF 2009 High-affinity binding of Chp1 chromodomain to K9 methylated histone H3 is required to establish centromeric heterochromatin. Mol Cell 34:36–46. doi:10.1016/j.molcel.2009.02.024. 19362535 PMC2705653

[30] Katan-Khaykovich Y, Struhl K 2005 Heterochromatin formation involves changes in histone modifications over multiple cell generations. EMBO J 24:2138–2149. doi:10.1038/sj.emboj.7600692. 15920479 PMC1150886

[31] Radman-Livaja M, Ruben G, Weiner A, Friedman N, Kamakaka R, Rando OJ 2011 Dynamics of Sir3 spreading in budding yeast: secondary recruitment sites and euchromatic localization. EMBO J 30:1012–1026. doi:10.1038/emboj.2011.30. 21336256 PMC3061035

[32] Martins-Taylor K, Dula Lou M, Holmes SG 2004 Heterochromatin spreading at yeast telomeres occurs in M phase. Genetics 168:65–75. doi:10.1534/genetics.103.020149. 15454527 PMC1448083

[33] Diede SJ, Gottschling DE 1999 Telomerase-mediated telomere addition in vivo requires DNA primase and DNA polymerases alpha and delta. Cell 99:723–733. doi:10.1016/S0092-8674(00)81670-0. 10619426

[34] Sunder S, Greeson-Lott NT, Runge KW, Sanders SL 2012 A new method to efficiently induce a site-specific double-strand break in the fission yeast Schizosaccharomyces pombe. Yeast 29:275–291. doi:10.1002/yea.2908. 22674789 PMC3389596

[35] Harrison JC, Haber JE 2006 Surviving the breakup: the DNA damage checkpoint. Annu Rev Genet 40:209–235. doi:10.1146/annurev.genet.40.051206.105231. 16805667

[36] Ribeyre C, Shore D 2012 Anticheckpoint pathways at telomeres in yeast. Nat Struct Mol Biol 19:307–313. doi:10.1038/nsmb.2225. 22343724

[37] Michelson RJ, Rosenstein S, Weinert T 2005 A telomeric repeat sequence adjacent to a DNA double-stranded break produces an anticheckpoint. Genes Dev 19:2546–2559. doi:10.1101/gad.1293805. 16230525 PMC1276729

[38] Lee SS, Bohrson C, Pike AM, Wheelan SJ, Greider CW 2015 ATM kinase is required for telomere elongation in mouse and human cells. Cell Rep 13:1623–1632. doi:10.1016/j.celrep.2015.10.035. 26586427 PMC4663052

[39] Ribeyre C, Shore D 2013 Regulation of telomere addition at DNA double-strand breaks. Chromosoma 122:159–173. doi:10.1007/s00412-013-0404-2. 23504035

[40] Wang J, Lawry ST, Cohen AL, Jia S 2014 Chromosome boundary elements and regulation of heterochromatin spreading. Cell Mol Life Sci 71:4841–4852. doi:10.1007/s00018-014-1725-x. 25192661 PMC4234687

[41] Watson AT, Werler P, Carr AM 2011 Regulation of gene expression at the fission yeast Schizosaccharomyces pombe urg1 locus. Gene 484:75–85. doi:10.1016/j.gene.2011.05.028. 21664261

[42] Grewal SS, Klar AJ 1997 A recombinationally repressed region between *mat2* and *mat3* loci shares homology to centromeric repeats and regulates directionality of mating-type switching in fission yeast. Genetics 146:1221–1238. 9258669 10.1093/genetics/146.4.1221 PMC1208070

[43] Thon G, Bjerling P, Bunner C, Verhein-Hansen J 2002 Expression-state boundaries in the mating-type region of fission yeast. Genetics 161:611–622. 12072458 10.1093/genetics/161.2.611 PMC1462127

[44] Wood V, Gwilliam R, Rajandream M-A, Lyne M, Lyne R, Stewart A, Sgouros J, Peat N, Hayles J, Baker S, Basham D, Bowman S, Brooks K, Brown D, Brown S, Chillingworth T, Churcher C, Collins M, Connor R, Cronin A, Davis P, Feltwell T, Fraser A, Gentles S, Goble A, Hamlin N, Harris D, Hidalgo J, Hodgson G, Holroyd S, Hornsby T, Howarth S, Huckle EJ, Hunt S, Jagels K, James K, Jones L, Jones M, Leather S, McDonald S, McLean J, Mooney P, Moule S, Mungall K, Murphy L, Niblett D, Odell C, Oliver K, O'Neil S, Pearson D, Quail MA, Rabbinowitsch E, Rutherford K, Rutter S, Saunders D, Seeger K, Sharp S, Skelton J, Simmonds M, Squares R, Squares S, Stevens K, Taylor K, Taylor RG, Tivey A, Walsh S, Warren T, Whitehead S, Woodward J, Volckaert G, Aert R, Robben J, Grymonprez B, Weltjens I, Vanstreels E, Rieger M, Schäfer M, Müller-Auer S, Gabel C, Fuchs M, Düsterhöft A, Fritzc C, Holzer E, Moestl D, Hilbert H, Borzym K, Langer I, Beck A, Lehrach H, Reinhardt R, Pohl TM, Eger P, Zimmermann W, Wedler H, Wambutt R, Purnelle B, Goffeau A, Cadieu E, Dréano S, Gloux S, Lelaure V, Mottier S, Galibert F, Aves SJ, Xiang Z, Hunt C, Moore K, Hurst SM, Lucas M, Rochet M, Gaillardin C, Tallada VA, Garzon A, Thode G, Daga RR, Cruzado L, Jimenez J, Sánchez M, del Rey F, Benito J, Domínguez A, Revuelta JL, Moreno S, Armstrong J, Forsburg SL, Cerutti L, Lowe T, McCombie WR, Paulsen I, Potashkin J, Shpakovski GV, Ussery D, Barrell BG, Nurse P, Cerrutti L 2002 The genome sequence of Schizosaccharomyces pombe. Nature 415:871–880. doi:10.1038/nature724. 11859360

[45] Cromie GA, Rubio CA, Hyppa RW, Smith GR 2005 A natural meiotic DNA break site in Schizosaccharomyces pombe is a hotspot of gene conversion, highly associated with crossing over. Genetics 169:595–605. doi:10.1534/genetics.104.037176. 15545638 PMC1449127

[46] Farah JA, Cromie GA, Smith GR 2009 Ctp1 and exonuclease 1, alternative nucleases regulated by the MRN complex, are required for efficient meiotic recombination. Proc Natl Acad Sci U S A 106:9356–9361. doi:10.1073/pnas.0902793106. 19470480 PMC2695049

[47] Forsburg SL 1994 Codon usage table for Schizosaccharomyces pombe. Yeast 10:1045–1047. doi:10.1002/yea.320100806. 7992504

[48] Watson AT, Daigaku Y, Mohebi S, Etheridge TJ, Chahwan C, Murray JM, Carr AM 2013 Optimisation of the Schizosaccharomyces pombe urg1 expression system. PLoS One 8:e83800. doi:10.1371/journal.pone.0083800. 24376751 PMC3869809

[49] Watt S, Mata J, López-Maury L, Marguerat S, Burns G, Bähler J 2008 urg1: a uracil-regulatable promoter system for fission yeast with short induction and repression times. PLoS One 3:e1428. doi:10.1371/journal.pone.0001428. 18197241 PMC2174524

[50] Li P, Li J, Li M, Dou K, Zhang M-J, Suo F, Du L-L 2012 Multiple end joining mechanisms repair a chromosomal DNA break in fission yeast. DNA Repair 11:120–130. doi:10.1016/j.dnarep.2011.10.011. 22093869

[51] Wood V, Harris MA, McDowall MD, Rutherford K, Vaughan BW, Staines DM, Aslett M, Lock A, Bähler J, Kersey PJ, Oliver SG 2012 PomBase: a comprehensive online resource for fission yeast. Nucleic Acids Res 40:D695–D699. doi:10.1093/nar/gkr853. 22039153 PMC3245111

[52] Wang X, Baumann P 2008 Chromosome fusions following telomere loss are mediated by single-strand annealing. Mol Cell 31:463–473. doi:10.1016/j.molcel.2008.05.028. 18722173

[53] Webb CJ, Zakian VA 2008 Identification and characterization of the Schizosaccharomyces pombe TER1 telomerase RNA. Nat Struct Mol Biol 15:34–42. doi:10.1038/nsmb1354. 18157149 PMC2703720

[54] Bairley RCB, Guillaume G, Vega LR, Friedman KL 2011 A mutation in the catalytic subunit of yeast telomerase alters primer-template alignment while promoting processivity and protein-DNA binding. J Cell Sci 124:4241–4252. doi:10.1242/jcs.090761. 22193961 PMC4074303

[55] Murray AW, Claus TE, Szostak JW 1988 Characterization of two telomeric DNA processing reactions in Saccharomyces cerevisiae. Mol Cell Biol 8:4642–4650. doi:10.1128/MCB.8.11.4642. 3062364 PMC365553

[56] Gao Q, Reynolds GE, Wilcox A, Miller D, Cheung P, Artandi SE, Murnane JP 2008 Telomerase-dependent and -independent chromosome healing in mouse embryonic stem cells. DNA Repair 7:1233–1249. doi:10.1016/j.dnarep.2008.04.004. 18502190 PMC2597172

[57] Wellinger RJ, Zakian VA 2012 Everything you ever wanted to know about Saccharomyces cerevisiae telomeres: beginning to end. Genetics 191:1073–1105. doi:10.1534/genetics.111.137851. 22879408 PMC3415994

[58] Lydeard JR, Lipkin-Moore Z, Jain S, Eapen VV, Haber JE 2010 Sgs1 and exo1 redundantly inhibit break-induced replication and de novo telomere addition at broken chromosome ends. PLoS Genet 6:e1000973. doi:10.1371/journal.pgen.1000973. 20523895 PMC2877739

[59] Fukunaga K, Hirano Y, Sugimoto K 2012 Subtelomere-binding protein Tbf1 and telomere-binding protein Rap1 collaborate to inhibit localization of the Mre11 complex to DNA ends in budding yeast. Mol Biol Cell 23:347–359. doi:10.1091/mbc.e11-06-0568. 22130795 PMC3258178

[60] Capasso H, Palermo C, Wan S, Rao H, John UP, O'Connell MJ, Walworth NC 2002 Phosphorylation activates Chk1 and is required for checkpoint-mediated cell cycle arrest. J Cell Sci 115:4555–4564. doi:10.1242/jcs.00133. 12415000

[61] Latif C, den Elzen N, O'Connel M 2004 DNA damage checkpoint maintenance through sustained Chk1 activity. J Cell Sci 117:3489–3498. doi:10.1242/jcs.01204. 15213253

[62] Grimm C, Kohli J, Murray J, Maundrell K 1988 Genetic engineering of Schizosaccharomyces pombe: a system for gene disruption and replacement using the ura4 gene as a selectable marker. Mol Gen Genet 215:81–86. doi:10.1007/BF00331307. 3241624

[63] Elgin SCR, Reuter G 2013 Position-effect variegation, heterochromatin formation, and gene silencing in Drosophila. Cold Spring Harb Perspect Biol 5:a017780. doi:10.1101/cshperspect.a017780. 23906716 PMC3721279

[64] Lee NN, Chalamcharla VR, Reyes-Turcu F, Mehta S, Zofall M, Balachandran V, Dhakshnamoorthy J, Taneja N, Yamanaka S, Zhou M, Grewal SIS 2013 XMtr4-like protein coordinates nuclear RNA processing for heterochromatin assembly and for telomere maintenance. Cell 155:1061–1074. doi:10.1016/j.cell.2013.10.027. 24210919 PMC3974623

[65] Chalamcharla VR, Folco HD, Dhakshnamoorthy J, Grewal SIS 2015 Conserved factor Dhp1/Rat1/Xrn2 triggers premature transcription termination and nucleates heterochromatin to promote gene silencing. Proc Natl Acad Sci U S A 112:15548–15555. doi:10.1073/pnas.1522127112. 26631744 PMC4697380

[66] Moser BA, Subramanian L, Khair L, Chang Y-T, Nakamura TM 2009 Fission yeast Tel1(ATM) and Rad3(ATR) promote telomere protection and telomerase recruitment. PLoS Genet 5:e1000622. doi:10.1371/journal.pgen.1000622. 19714219 PMC2726628

[67] Harland JL, Chang YT, Moser BA, Nakamura TM 2014 Tpz1-Ccq1 and Tpz1-Poz1 interactions within fission yeast shelterin modulate Ccq1 Thr93 phosphorylation and telomerase recruitment. PLoS Genet 10:e1004708. doi:10.1371/journal.pgen.1004708. 25330395 PMC4199508

[68] Webb CJ, Zakian VA 2012 Schizosaccharomyces pombe Ccq1 and TER1 bind the 14-3-3-like domain of Est1, which promotes and stabilizes telomerase-telomere association. Genes Dev 26:82–91. doi:10.1101/gad.181826.111. 22215813 PMC3258969

[69] Webb CJ, Zakian VA 2015 Telomerase RNA stem terminus element affects template boundary element function, telomere sequence, and shelterin binding. Proc Natl Acad Sci U S A 112:11312–11317. doi:10.1073/pnas.1503157112. 26305931 PMC4568702

[70] Allshire RC, Javerzat JP, Redhead NJ, Cranston G 1994 Position effect variegation at fission yeast centromeres. Cell 76:157–169. doi:10.1016/0092-8674(94)90180-5. 8287474

[71] Sabatier L, Ricoul M, Pottier G, Murnane JP 2005 The loss of a single telomere can result in instability of multiple chromosomes in a human tumor cell line. Mol Cancer Res 3:139–150. doi:10.1158/1541-7786.MCR-04-0194. 15798094

[72] Yu S, Graf WD 2010 Telomere capture as a frequent mechanism for stabilization of the terminal chromosomal deletion associated with inverted duplication. Cytogenet Genome Res 129:265–274. doi:10.1159/000315887. 20606397

[73] Yatsenko SA, Brundage EK, Roney EK, Cheung SW, Chinault AC, Lupski JR 2009 Molecular mechanisms for subtelomeric rearrangements associated with the 9q34.3 microdeletion syndrome. Hum Mol Genet 18:1924–1936. doi:10.1093/hmg/ddp114. 19293338 PMC2678925

[74] Osborne EA, Hiraoka Y, Rine J 2011 Symmetry, asymmetry, and kinetics of silencing establishment in Saccharomyces cerevisiae revealed by single-cell optical assays. Proc Natl Acad Sci U S A 108:1209–1216. doi:10.1073/pnas.1018742108. 21262833 PMC3029714

[75] Hathaway NA, Bell O, Hodges C, Miller EL, Neel DS, Crabtree GR 2012 Dynamics and memory of heterochromatin in living cells. Cell 149:1447–1460. doi:10.1016/j.cell.2012.03.052. 22704655 PMC3422694

[76] Klar AJS, Ishikawa K, Moore S 2014 A unique DNA recombination mechanism of the mating/cell-type switching of fission yeasts: a review. Microbiol Spectr 2:1–16. doi:10.1128/microbiolspec.MDNA3-0003-2014. PMC7687047 26104357

[77] Martienssen R, Moazed D 2015 RNAi and heterochromatin assembly. Cold Spring Harb Perspect Biol 7:a019323. doi:10.1101/cshperspect.a019323. 26238358 PMC4526745

[78] Allshire RC, Ekwall K 2015 Epigenetic regulation of chromatin states in Schizosaccharomyces pombe Cold Spring Harb Perspect Biol 7:a018770. doi:10.1101/cshperspect.a018770. 26134317 PMC4484966

[79] Kageyama S, Liu H, Kaneko N, Ooga M, Nagata M, Aoki F 2007 Alterations in epigenetic modifications during oocyte growth in mice. Reproduction 133:85–94. doi:10.1530/REP-06-0025. 17244735

[80] Ge Z-J, Schatten H, Zhang C-L, Sun Q-Y 2015 Oocyte ageing and epigenetics. Reproduction 149:R103–E114. doi:10.1530/REP-14-0242. 25391845 PMC4397590

[81] Moreno S, Klar A, Nurse P 1991 Molecular genetic analysis of fission yeast Schizosaccharomyces pombe. Methods Enzymol 194:795–823. doi:10.1016/0076-6879(91)94059-L. 2005825

[82] Reference deleted.

[83] Erler A, Maresca M, Fu J, Stewart AF 2006 Recombineering reagents for improved inducible expression and selection marker re-use in Schizosaccharomyces pombe. Yeast 23:813–823. doi:10.1002/yea.1396. 16921581

[84] Muhlrad D, Hunter R, Parker R 1992 A rapid method for localized mutagenesis of yeast genes. Yeast 8:79–82. doi:10.1002/yea.320080202. 1561838

[85] Ray A, Runge KW 1999 The yeast telomere length counting machinery is sensitive to sequences at the telomere-nontelomere junction. Mol Cell Biol 19:31–45. doi:10.1128/MCB.19.1.31. 9858529 PMC83863

[86] Hector RE, Ray A, Chen B-R, Shtofman R, Berkner KL, Runge KW 2012 Mec1p associates with functionally compromised telomeres. Chromosoma 121:277–290. doi:10.1007/s00412-011-0359-0. 22289863 PMC3350766

[87] Förstemann K, Höss M, Lingner J 2000 Telomerase-dependent repeat divergence at the 3′ ends of yeast telomeres. Nucleic Acids Res 28:2690–2694. doi:10.1093/nar/28.14.2690. 10908324 PMC102662

[88] Moser BA, Chang Y, Nakamura TM 2014 Telomere regulation during the cell cycle in fission yeast. Methods Mol Biol 1170:411–424. doi:10.1007/978-1-4939-0888-2_22. 24906327 PMC4469975

[89] Fisher TS, Taggart AKP, Zakian VA 2004 Cell cycle-dependent regulation of yeast telomerase by Ku. Nat Struct Mol Biol 11:1198–1205. doi:10.1038/nsmb854. 15531893

[90] Kiely CM, Marguerat S, Garcia JF, Madhani HD, Bähler J, Winston F 2011 Spt6 is required for heterochromatic silencing in the fission yeast Schizosaccharomyces pombe. Mol Cell Biol 31:4193–4204. doi:10.1128/MCB.05568-11. 21844224 PMC3187285

[91] Kato H, Okazaki K, Iida T, Nakayama J-I, Murakami Y, Urano T 2013 Spt6 prevents transcription-coupled loss of posttranslationally modified histone H3. Sci Rep 3:2186. doi:10.1038/srep02186. 23851719 PMC3711048

[92] Yamada S, Ohta K, Yamada T 2013 Acetylated histone H3K9 is associated with meiotic recombination hotspots, and plays a role in recombination redundantly with other factors including the H3K4 methylase Set1 in fission yeast. Nucleic Acids Res 41:3504–3517. doi:10.1093/nar/gkt049. 23382177 PMC3616738

[93] Oya E, Kato H, Chikashige Y, Tsutsumi C, Hiraoka Y, Murakami Y 2013 Mediator directs co-transcriptional heterochromatin assembly by RNA interference-dependent and -independent pathways. PLoS Genet 9:e1003677. doi:10.1371/journal.pgen.1003677. 23966866 PMC3744440

[94] Kramer KM, Haber JE 1993 New telomeres in yeast are initiated with a highly selected subset of TG1-3 repeats. Genes Dev 7:2345–2356. doi:10.1101/gad.7.12a.2345. 8253381

